# Situational Awareness in Telehealth: A Virtual Standardized Patient Case for Transitioning Preclinical to Clinical Medical Students

**DOI:** 10.15766/mep_2374-8265.11517

**Published:** 2025-04-11

**Authors:** Patricia Pozo, Marianfeli Cecilia Landino, Joni M. Maga, Lydia M. Jorge, Roxanna Araya, Danielle Bodzin Horn, Alecia L. S. Stein

**Affiliations:** 1 Fourth-Year Medical Student, University of Miami Leonard M. Miller School of Medicine; 2 Research Support Specialist, University of Miami/Jackson Memorial Hospital Center for Patient Safety; 3 Associate Professor, Department of Anesthesiology, Perioperative Medicine and Pain Management, University of Miami/Jackson Memorial Hospital Center for Patient Safety; 4 Assistant Professor, Department of Anesthesiology, Perioperative Medicine and Pain Management, University of Miami Leonard M. Miller School of Medicine; 5 Simulation Training Specialist, University of Miami/Jackson Memorial Hospital Center for Patient Safety; 6 Assistant Professor, Department of Anesthesiology, Perioperative Medicine and Pain Management, University of Miami Leonard M. Miller School of Medicine; 7 Associate Professor, Department of Anesthesiology, Perioperative Medicine and Pain Management, University of Miami/Jackson Memorial Hospital Center for Patient Safety

**Keywords:** Communication Errors, Situational Awareness, Communication Skills, Simulation, Standardized Patient, Telehealth

## Abstract

**Introduction:**

Since the COVID-19 pandemic, telehealth usage has surged and remains in high demand even as public health restrictions have relaxed. Telehealth introduces unique challenges to patient care, particularly in maintaining situational awareness and patient safety. Despite telehealth's widespread use, its training is still underrepresented in medical curricula. Recognizing these gaps, we developed a focused module to teach medical students about the significant differences between virtual and in-person visits, emphasizing essential communication skills and tools for situational awareness through simulated telehealth consultations.

**Methods:**

Using a virtual platform, second-year (preclinical) medical students participated in a simulated telehealth visit during which they conducted a focused assessment of a standardized patient. While the chief complaint was a generalized symptom, the standardized patient had characteristics that suggested a more critical problem that would only be recognized with maintenance of appropriate situational awareness. Faculty members observed the students’ performance and provided brief group feedback and discussion along with input from the standardized professionals.

**Results:**

Over 2 years, 634 preclinical medical students completed the simulated telehealth session during a course dedicated to transitioning students to their clinical rotations. Feedback from more than 80% of the medical students indicated that the training was valuable and exceeded their expectations.

**Discussion:**

Our innovative simulated telehealth consultation effectively enhances medical students’ knowledge and proficiency in telehealth practices and situational awareness, providing them with essential skills for the evolving health care landscape.

## Educational Objectives

By the end of this activity, learners will be able to:

1. Outline key components of a typical telehealth encounter.
2. List effective strategies to reduce communication errors in the telehealth clinical environment.
3. Demonstrate a focused patient assessment, including assurance of proper patient, setting, and confidentiality during a telehealth visit.
4. Explain the vital components of active situational awareness in the telehealth clinical environment.

## Introduction

Telehealth is a rapidly growing health care modality aimed at improving patient access, efficiency, and convenience and reducing health care disparities and costs. The COVID-19 pandemic accelerated the implementation of telehealth as health care professionals searched for means by which patients could interact with their physicians in a socially distanced setting. Notably, telehealth visits increased by 154% in the thirteenth week of 2020 when compared with the same week in 2019.^1^ Evidence demonstrates that this trend is not slowing.^2^ In fact, it is being widely embraced by both patients and clinicians alike,^3^ with 66% of patients saying they want the continued option of telehealth.^4^

Despite this increased usage, a study in 2023 showed that while most students can gather information and counsel patients via telehealth, their confidence significantly drops compared to in-person care.^5^ Recognizing a gap in medical students’ training regarding telehealth patient encounters, in 2021 our institution added a 50-minute telehealth patient module to our preexisting mandatory Transition to Clerkships Boot Camp educational activity. Our simulated telehealth encounter utilized a low-stakes formative role-playing methodology without formal assessment. This allowed preclinical students to assess highly trained standardized patients (SPs) via the SP program at the University of Miami.

Situational awareness (SA) is defined as “the perception of elements of the environment within a volume of time and space, the comprehension of their meaning and the projection of their status in the near future.”^6^ With respect to health care, providers must master novel technology, remain current in the literature, and, most importantly, adapt during the clinical interview.^7^ Current literature has identified three levels of SA.^8^ Level 1 involves perceiving environmental cues and collecting diagnostic information, level 2 requires comprehending this information to assess the situation accurately,^7^ and level 3 involves planning clinical outcomes by predicting future events for effective treatment. SA necessitates the entire health care team's vigilance to avoid threats to patient safety.

Numerous scholarly publications have focused on simulations for the telehealth education of medical students,^9–13^ nursing students,^14^ residents,^15^ and attending physicians.^16^ Many of these simulations have focused on comprehensive telehealth etiquette training, ranging from conducting thorough virtual physical examinations to optimizing lighting and camera adjustments.^9^ Wilson and colleagues created a simulated virtual geriatric patient encounter focusing on the specific challenges of telehealth for this vulnerable population, such as unfamiliarity with technology, cognitive and sensory barriers, and inclusion of caregivers.^13^ Other studies have forgone simulation entirely and instead embraced a group discussion approach. For example, Wang, Goodrich, Strauss, and Martindale designed a session that included a mix of small-group discussion and video demonstration.^17^ Notably, despite the absence of a simulated encounter, there was still a reported increase in proficiency with telehealth technology.^17^ Of the papers that have implemented simulations, many have focused on patient management via a virtual format rather than on the modality of telehealth itself, for example, menopause and pediatric case triaging.^10,18^ Some studies’ modules have delved into sensitive conversations and establishing patient trust, with a notable example involving a virtual psychiatric assessment for a depressed patient with suicidality.^11^

The inclusion of patient safety, specifically SA, as a curricular objective in telemedicine education has not yet been described in the literature. Given that communication errors contribute significantly to unexpected adverse events in patient care^19,20^ prioritizing this focus is essential for comprehensive telehealth education. Our primary objective was to teach medical students the standards and nuances of telehealth patient encounters, as well as how to maintain SA and enhance their skills for effective professional communication during telehealth visits. Our secondary objective was to assess medical student satisfaction and the value of the module based on self-reported feedback.

## Methods

### Session Design

The virtual nature of this module allowed simultaneous sessions with multiple breakout rooms and scenarios running in parallel, limited only by the availability of required personnel. To support psychological safety with respect to confidentiality, the sessions were not recorded. This educational module was reviewed and exempted (# 20231344, June 18, 2024) by the University of Miami Institutional Review Board.

### Materials

Materials for the module included the following:

• Student prework: telehealth guidelines (Appendix A)
• Faculty training guide (Appendix B)
• Simulated patient Melanie/Michael Jones scenario (Appendix C)
• SP survey tool (Appendix D)
• Scenario stem (Appendix E)
• Prebriefing presentation for students (Appendix F)
• Session facilitators presentation (Appendix G)
• Postencounter student survey (Appendix H)
• License for a virtual platform, such as Zoom

### Personnel

The ideal minimum number of personnel included the following:

• One course director
• One course coordinator
• Two session facilitators, one per Zoom link
• Two faculty debriefers, one per Zoom link
• Six SPs, three per Zoom link

### Session Preparation

In the weeks before the course launch, the schedule was finalized, faculty members were recruited, SPs were reserved, and personnel training was completed. The number of personnel required was dictated by the number of students, session duration, and number of necessary Zoom links. To accommodate 200 preclinical second-year medical students, we conducted two simultaneous sessions in separate Zoom rooms from 09:00 to 16:00 over 3 days. One week before the course, we updated the boot camp course schedule, assigning each student their session date, time, Zoom link, and prework, including a slide deck reviewing an overview of telehealth best practices and session instructions and objectives (Appendix A). SA was excluded from the students’ prework as alerting them to it before the session could have reduced the impact of this learning opportunity.

#### Faculty recruitment and training

The course director recruited 10 medical school faculty for a 1-hour virtual training, which was recorded and shared. The director reviewed the training schedule flowchart (Figure) and the faculty guide (Appendix B), which included debriefing points, strategies for integrating SP feedback into the debriefing, and methods to sustain student interaction. Faculty were also provided with the SP scenario (Appendix C) to review and familiarize themselves with the simulation.

**Figure.**
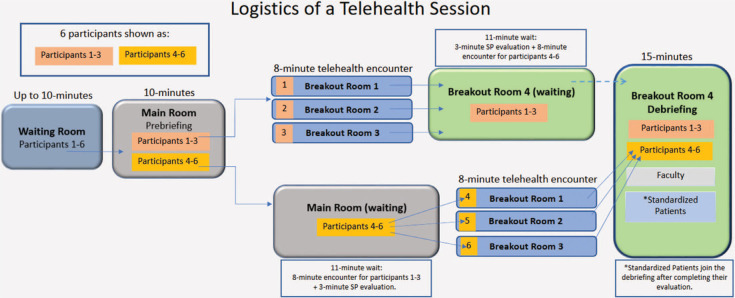
Logistics of a telehealth session: This flowchart outlines the day-of organization of the virtual telehealth encounter. Abbreviation: SP, standardized patient.

#### Personnel recruitment and training

The course coordinator reserved 10 SPs from the school's standardized participant program and conducted two 1-hour remote presentations with the SPs and facilitators.

Topics covered in the SP training included an overview of the Melanie/Michael Jones scenario, desired mood/affect from the SPs, videoconferencing etiquette, and moulage application instructions for the SPs (Appendix C). The SPs were provided with the SP survey tool to be used later to evaluate the students (Appendix D). The SPs were given a daily training schedule and a review of the virtual videoconferencing platform. They were also provided with the scenario stem (Appendix E).

Topics covered in the session facilitator training included an overview of videoconferencing etiquette and a detailed review of videoconferencing platform functions. Facilitators were provided with the prebriefing presentation for students (Appendix F), the session facilitators presentation (Appendix G), the scenario stem/door note (Appendix E), and the post encounter student survey (Appendix H). They were given a daily training schedule and the student rosters for their assigned date.

### Day-of Logistics

On the session day, all activities occurred via Zoom. The facilitator navigated transitions between the waiting room, main room, and four breakout rooms. The main room was used for the prebrief. Breakout rooms 1–3 hosted the simulated telehealth encounters, each with a designated SP. Debriefing was completed in breakout room 4. After the prebrief, the facilitator moved students 1–3 to breakout rooms 1–3 for their 8-minute telehealth encounters and then transferred them to wait in breakout room 4. Students 4–6 then rotated through breakout rooms 1–3 for their 8-minute telehealth encounters. Upon completion, all participants (students 1–6, faculty, and SPs) joined breakout room 4 for debriefing. This setup was mirrored in the second Zoom link, allowing up to 12 students per hour to complete the module (Figure).

#### Prebrief

The students were held in the virtual waiting room 10 minutes before their scheduled encounter. At the scheduled time, they were admitted to the main room for a brief orientation during which the facilitator presented a slideshow (with an attendance QR code) establishing psychological safety and presenting the case scenario and session organization (Appendix F).

#### Intrasession

Students 1–3 were invited to their respective breakout rooms, where the SP displayed the case stem (Appendix E) via screen share for a 30-second review, after which the SP stopped the screen share and the 8-minute encounter began. In at least one of the three breakout rooms, a faculty observer silently observed with audio and video disabled. This allowed them to assess the overall dynamics and performance of some of the students, permitting more informed feedback during the debrief.

### Postsession Events

#### SP evaluation

At the conclusion of the telehealth consultation, the students were transitioned from breakout rooms 1–3 to breakout room 4. The SPs were then allotted 3 minutes to complete a brief survey to evaluate their student (Appendix D). The entire process was repeated for students 4–6. The survey did not include any identifying information. These data were used internally to assess overall student performance for this project and were not intended for individualized feedback.

#### Debriefing

The faculty member initiated the debriefing session with six participants and three SPs in the debriefing room. Given that all participants were at the same introductory level, generalized feedback that addressed common themes and areas for improvement was provided. The SPs kept their sunglasses on until prompted to remove them (per the faculty debriefing guide within Appendix B). The session began by gauging the medical students’ initial reactions to the SP interaction. The discussion then covered several key topics: verifying patient identity, addressing the chief complaint, using contextual clues for differential diagnosis with poor historians, gathering a focused history, ensuring clear communication in telehealth, building rapport, overcoming social barriers like hesitation to ask about the sunglasses, reviewing the definition of SA and how to implement it, addressing uncomfortable topics such as scars or safety concerns, and planning next steps, including possible in-person follow-up.

#### Medical student evaluation

Following completion of the course, the course coordinator instructed the medical students to fill out an evaluation assessing their experience (Appendix H). This evaluation, which used a 5-point Likert scale (1 = *strongly disagree,* 5 = *strongly agree*) and had a free-text option, was our primary method to determine whether our objectives had been met.

## Results

### Participant Demographics

Over 2 years, 634 preclinical medical students participated in the telehealth module, distributed across three separate cohorts. In June 2021, 206 medical students participated. In September 2021 and September 2022, 211 and 217 medical students participated, respectively.

### SP Survey Questionnaire

The SPs submitted assessments of 538 students (84% participation). Ninety-two percent of students introduced themselves, and 83% confirmed the SPs’ names. Eighty-two percent of students neglected to request that the SP remove their sunglasses, and 92% of students did not inquire about the facial lesion. In 2022, an additional question asked whether students provided a follow-up plan, with 52% of the cohort doing so.

### Medical Student Postcourse Evaluation

Eighty-six percent of participating medical students provided feedback after the telehealth encounter course. In June 2021, 206 medical students who participated in the telehealth program (88%) agreed that the training was valuable for their transition to clinical rotations. In September 2021 and September 2022, 89% of participants agreed that they were able to identify key components of a typical telehealth patient encounter after completing the case scenario. Ninety-four percent agreed that the telehealth patient encounter and associated faculty debriefing helped them realize the importance of maintaining SA during the patient encounter. Furthermore, 89% agreed that they felt attuned to recognizing patient-specific characteristics that seemed out of context in telehealth patient encounters, and 87% felt better prepared to address uncomfortable moments with telehealth patients.

Medical students provided feedback indicating that the telehealth encounter was one of their favorite and most valuable activities during the boot camp week. Other positive comments included the following:

• “Telehealth encounter was very useful.”
• “The telehealth encounter was a great and helpful session.”
• “[The telehealth encounter] made me realize the importance of taking a second to observe and take in the scene of the patient without letting the chart and expectations of the interaction guide actions.”
• “The telehealth encounter was a valuable experience and exceeded my expectations. The doctors and actors made me think differently about prioritization of information and situational awareness.”

## Discussion

To our knowledge, this simulation module is the first to address both SA and basic telehealth skills. Patient safety was prioritized through key measures, such as confirming patient identification, ensuring a well-lit setting, and verifying the patient's privacy. We introduced the concept of SA as a vital component of patient safety using a simulated telehealth visit. This module effectively taught medical students how to maintain SA in a virtual environment, a critical skill for ensuring patient safety.^7^

Few students asked the SP to remove their sunglasses (18%), and even fewer asked about the patient's facial lesion (8%). During the debrief, the students noted various reasons for not addressing these important cues, which included the following: Some did not notice the lesion, some thought that the lesion and/or glasses were idiosyncratic to the SP and not a part of the simulation, some felt this was not a part of the focused history, some felt uncomfortable or underqualified to ask about the lesion, and others mentioned they did not have enough time. Students who did not address the glasses and/or lesion missed critical information leading to a potential skin cancer diagnosis. Furthermore, the importance of adopting a professional mindset despite the unnatural virtual environment was emphasized during the group debriefs, specifically the importance of putting aside social norms and sensitivities to address potentially embarrassing or uncomfortable issues with the patient, as well as how to present next steps (i.e., transitioning care to a near-future in-person visit). This simulation accomplished our desired goals and revealed pathways for future educational scholarly work as we determined that preclinical students do not routinely employ aspects of SA.

Staffing was a significant challenge, particularly the need for two session facilitators in addition to SPs for each Zoom meeting. For the sake of efficiency, two Zoom meetings were conducted simultaneously. Additional faculty staffing would enable more direct observation in every room, allowing for more specific feedback. Technical issues with Zoom also presented challenges, highlighting the necessity of having a specified coordinator to guide the movement of students and facilitators through the rooms (i.e., from the waiting room to the main room to the breakout rooms for the patient encounter and finally to the debriefing room) to improve flow and organization. Staff observations and student feedback indicated that the 8-minute encounter time was sufficient for a quality patient interaction.

Trainees were evaluated during and immediately following the encounter, with no long-term follow-up after the encounter to directly assess whether the proposed objectives were met in actual telehealth practice. Additionally, the self-reflection form sent out to participants after the encounter was optional, which resulted in limited feedback about satisfaction with and efficacy of the simulation and introduced voluntary response bias. This project may be challenging to replicate at an institution without an established SP program such as the one at ours. Our SPs received minimal training, requiring only one brief meeting after written instructions were sent out. The simulation would have been less efficient without access to highly trained SPs.

Future directions for our telehealth module include continuing the encounter for preclinical students during their Transition to Clerkships Boot Camp course before beginning clerkships. In future years, we will vary the physical presentation of the SPs; for example, instead of a mole, we will have the SP present with a bruise to open the conversation to alternative important yet uncomfortable questions. Six months following completion of the module, a subsequent simulation could be conducted to allow students to implement these learned SA skills and to assess whether their SA has improved following this activity. A follow-up survey to obtain self-reported feedback could be distributed to the participants at the end of their clinical rotation year to gauge whether they perceive that the module provided sustained improvement in their ability to conduct real telehealth encounters and to implement the principles of SA in their clinical practice.

## Appendices


Student Prework.pptxFaculty Training Guide.docxSP Scenario.docxSP Survey Tool.docxScenario Stem.pptxStudent Prebriefing.pptxSession Facilitators Presentation.pptxPostencounter Student Survey.docx
*All appendices are peer reviewed as integral parts of the Original Publication.*
