# Supplementary material for: Situational Awareness in Telehealth: A Virtual Standardized Patient Case for Transitioning Preclinical to Clinical Medical Students
Source: MedEdPORTAL. 2025 Apr 11;21:11517. doi: 10.15766/mep_2374-8265.11517 (PMC11985545; doi:10.15766/mep_2374-8265.11517)
Supplement: Supplementary file 1 — Student Prework.pptxFaculty Training Guide.docxSP Scenario.docxSP Survey Tool.docxScenario Stem.pptxStudent Prebriefing.pptxSession Facilitators Presentation.pptxPostencounter Student Survey.docx [file mep_2374-8265.11517-s001.zip › A. Student Prework.pptx]

## Slide 1
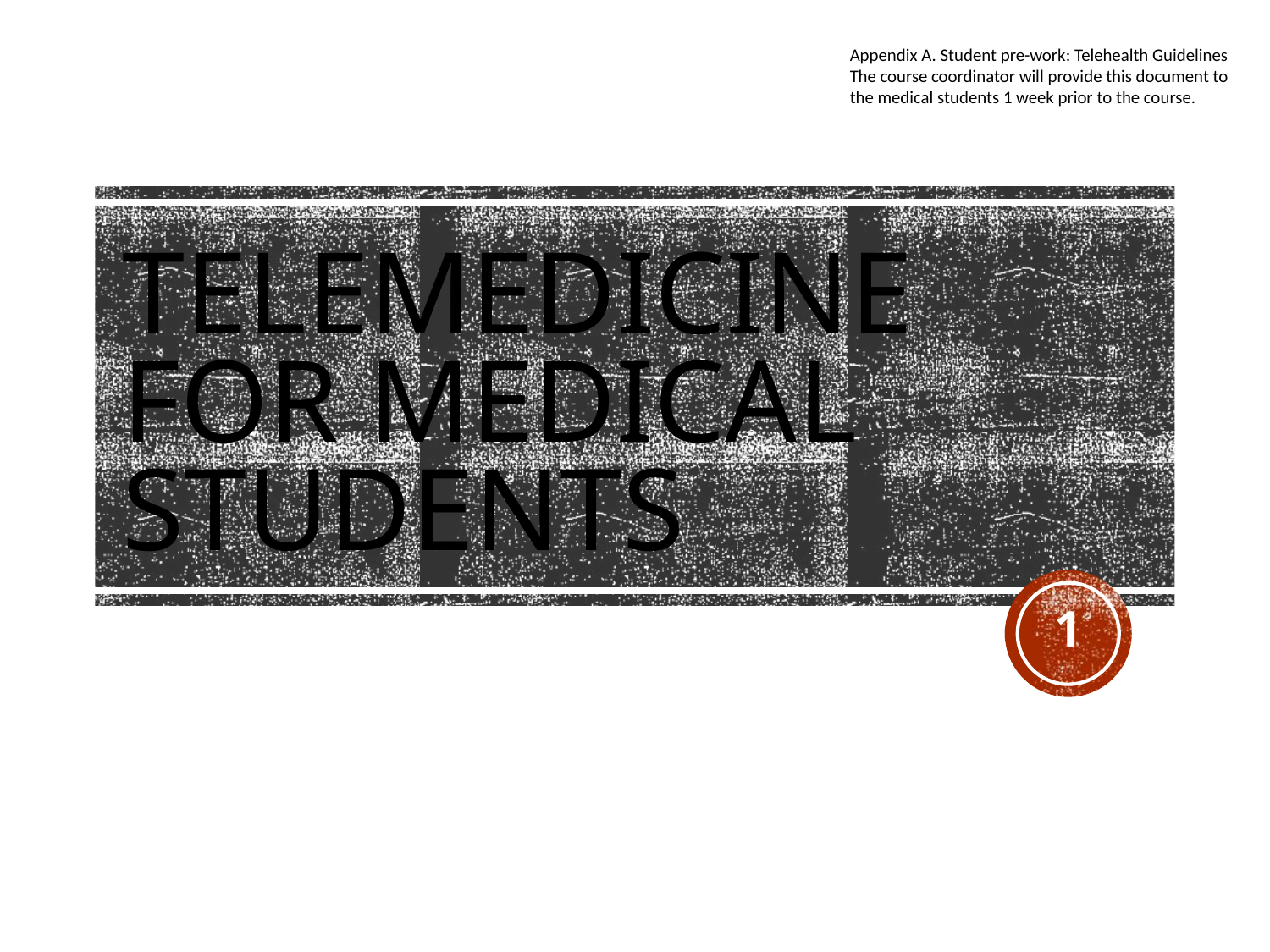

Appendix A. Student pre-work: Telehealth Guidelines
The course coordinator will provide this document to the medical students 1 week prior to the course.
# Telemedicine for Medical Students
1

## Slide 2
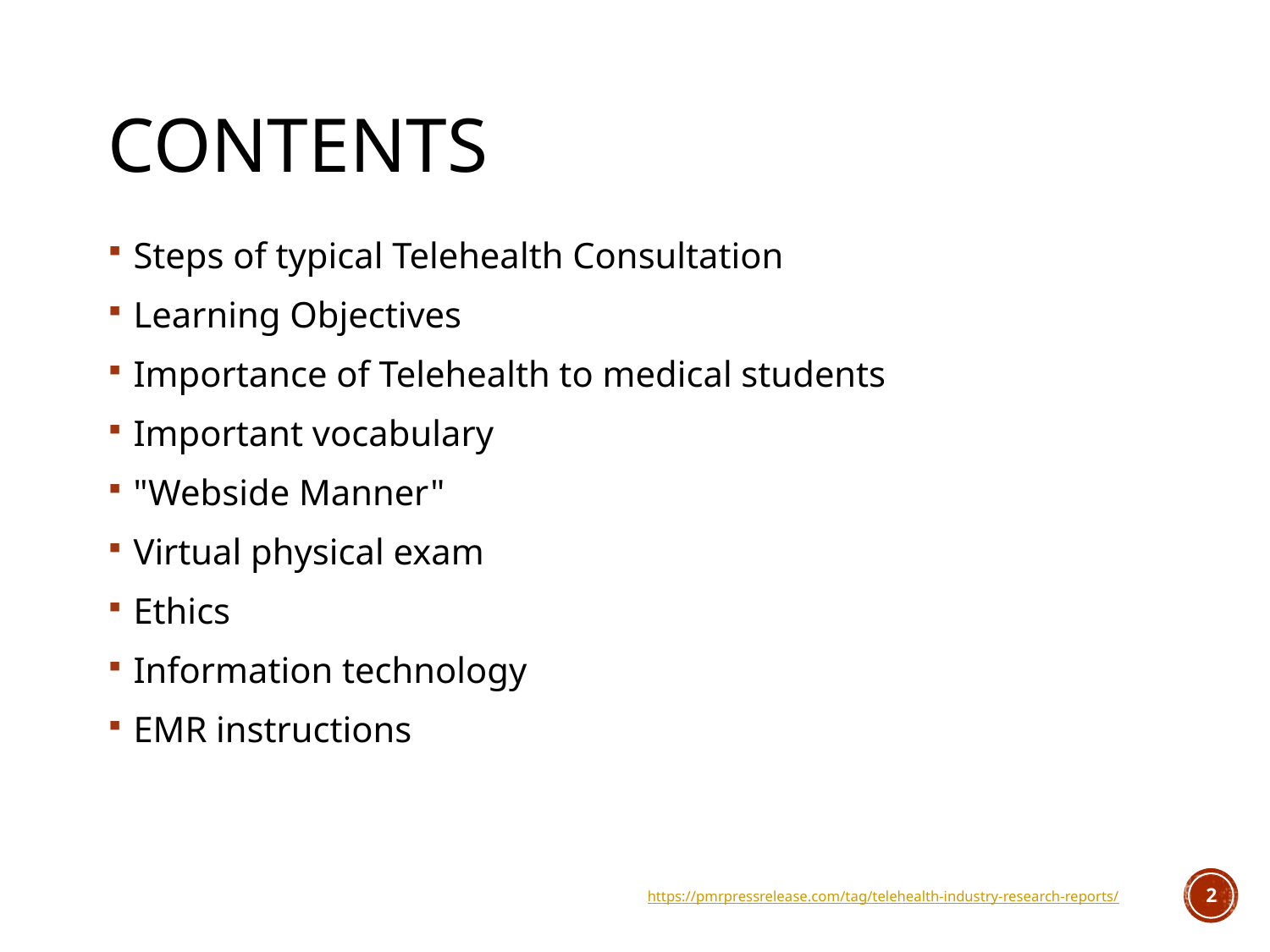

# Contents
Steps of typical Telehealth Consultation
Learning Objectives
Importance of Telehealth to medical students
Important vocabulary
"Webside Manner"
Virtual physical exam
Ethics
Information technology
EMR instructions
2
https://pmrpressrelease.com/tag/telehealth-industry-research-reports/

## Slide 3
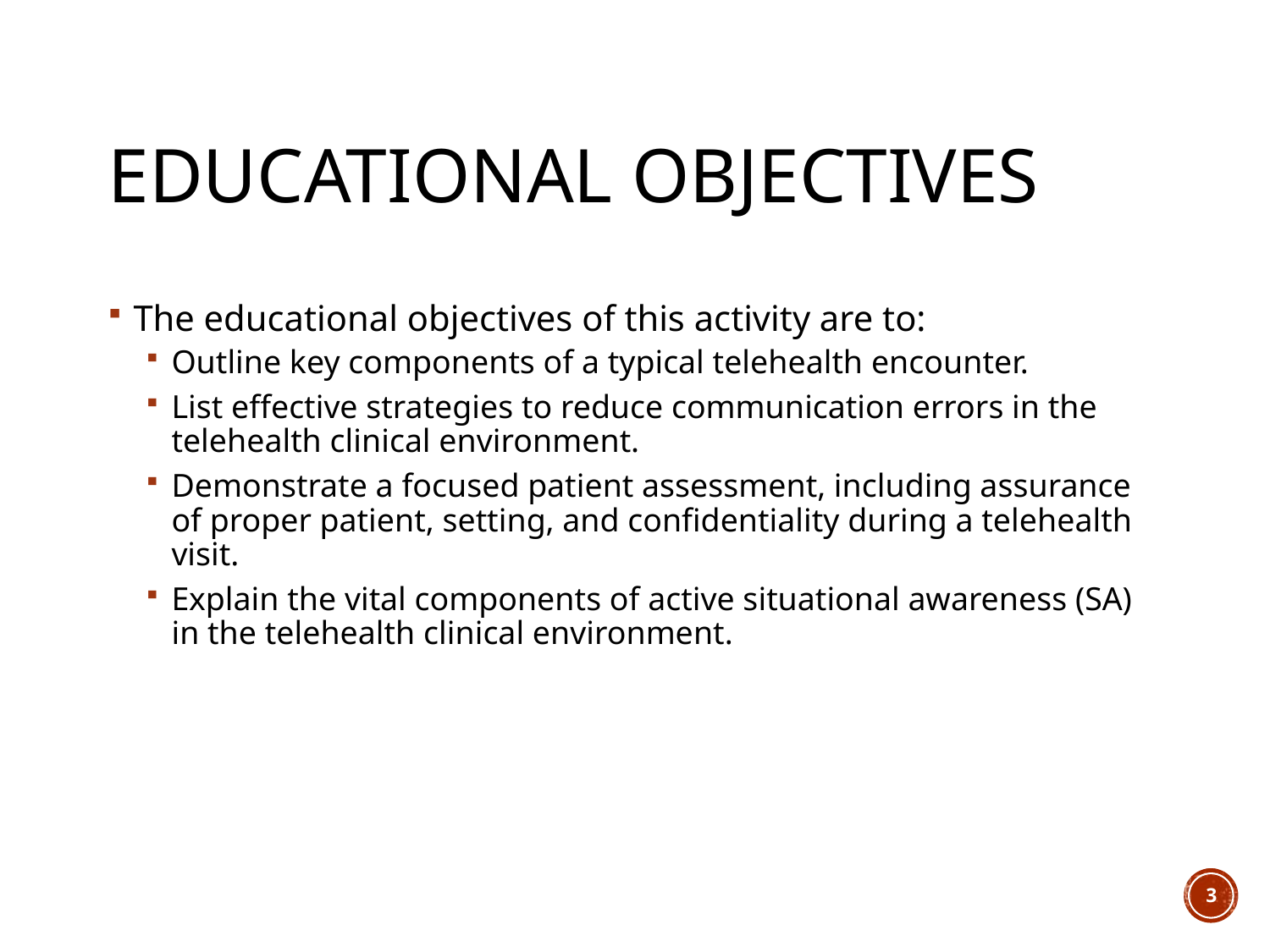

# Educational Objectives
The educational objectives of this activity are to:
Outline key components of a typical telehealth encounter.
List effective strategies to reduce communication errors in the telehealth clinical environment.
Demonstrate a focused patient assessment, including assurance of proper patient, setting, and confidentiality during a telehealth visit.
Explain the vital components of active situational awareness (SA) in the telehealth clinical environment.
3

## Slide 4
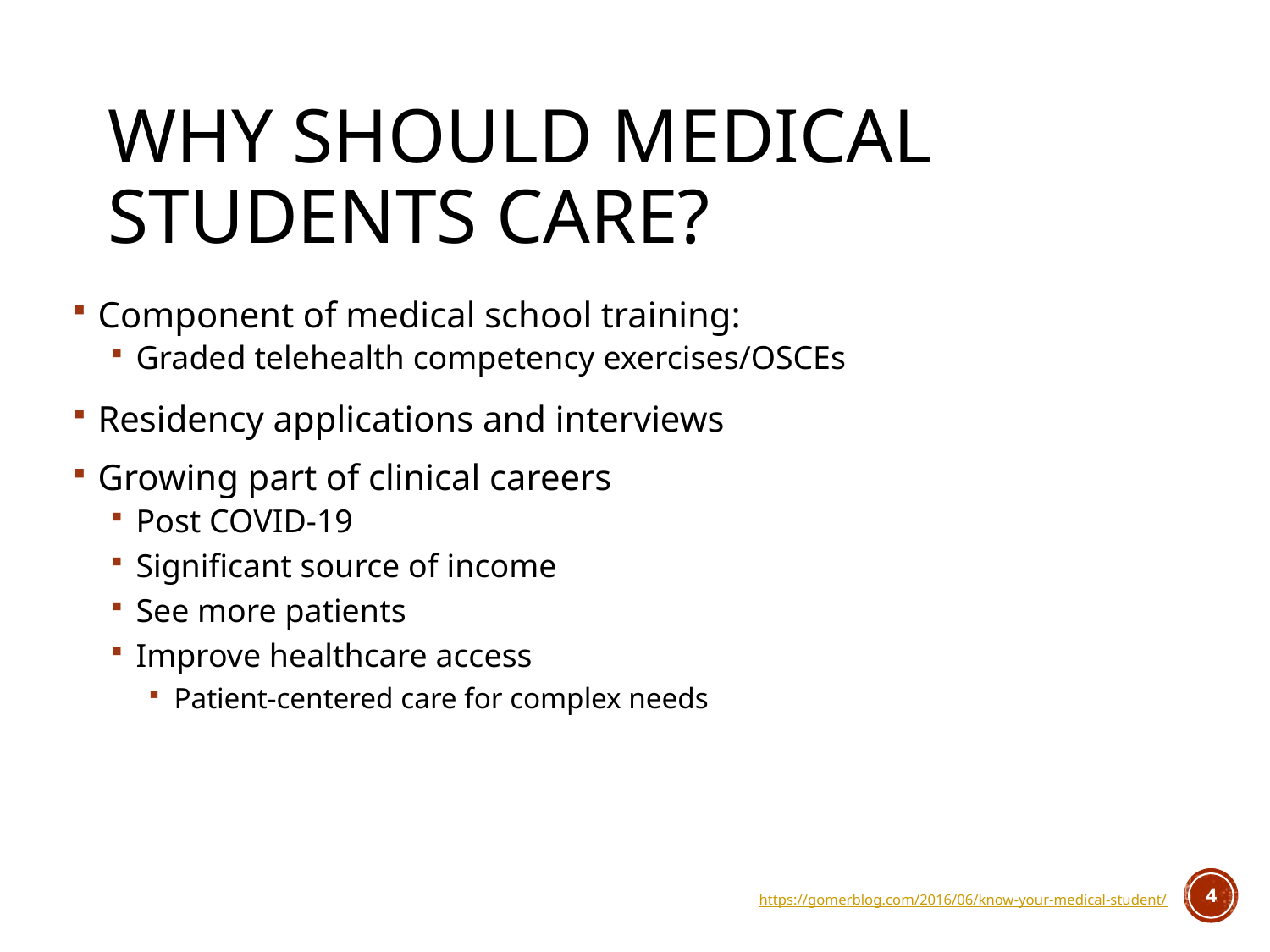

# Why Should Medical Students Care?
Component of medical school training:
Graded telehealth competency exercises/OSCEs
Residency applications and interviews
Growing part of clinical careers
Post COVID-19
Significant source of income
See more patients
Improve healthcare access
Patient-centered care for complex needs
4
https://gomerblog.com/2016/06/know-your-medical-student/

## Slide 5
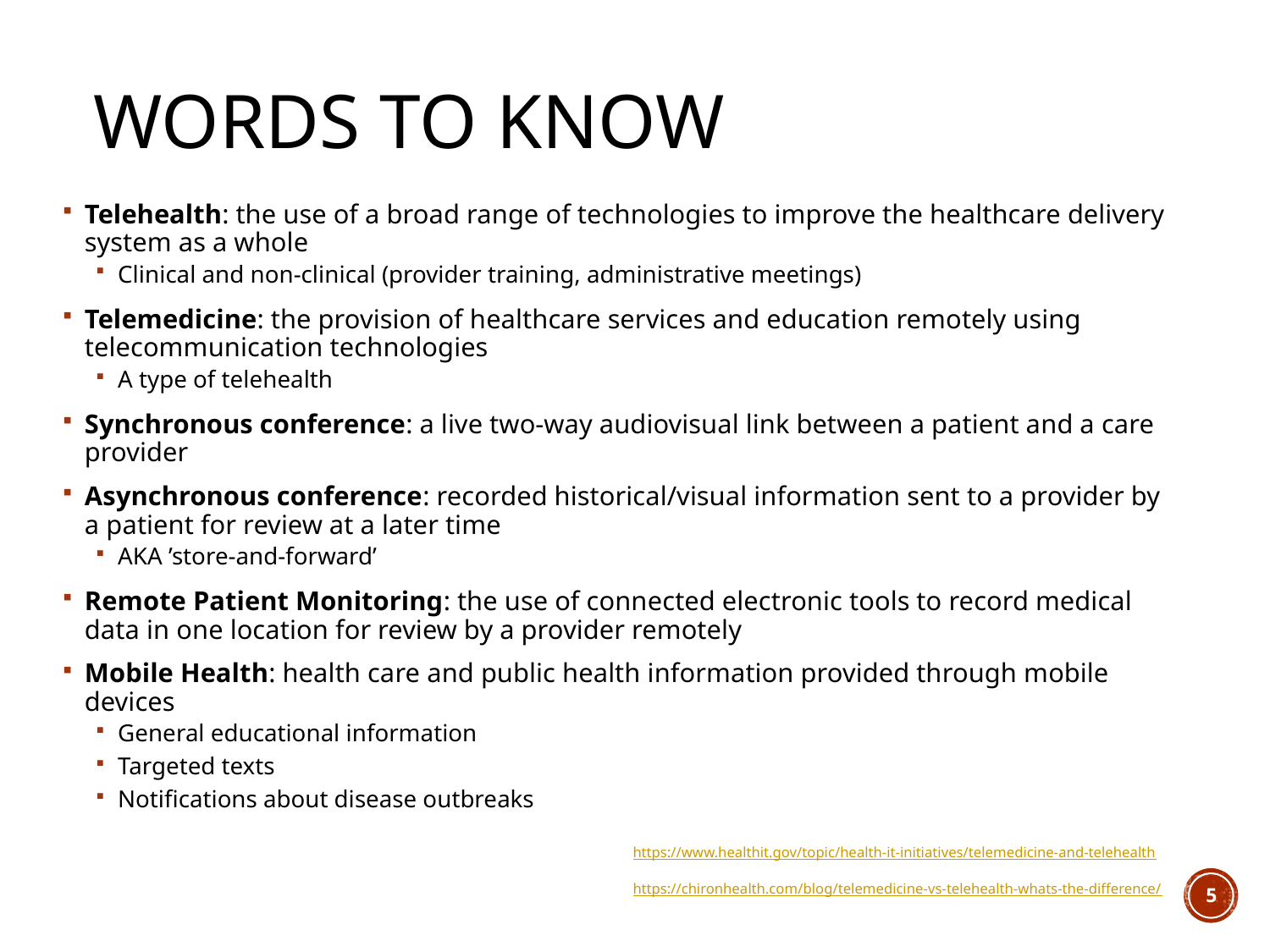

# Words to know
Telehealth: the use of a broad range of technologies to improve the healthcare delivery system as a whole
Clinical and non-clinical (provider training, administrative meetings)
Telemedicine: the provision of healthcare services and education remotely using telecommunication technologies
A type of telehealth
Synchronous conference: a live two-way audiovisual link between a patient and a care provider
Asynchronous conference: recorded historical/visual information sent to a provider by a patient for review at a later time
AKA ’store-and-forward’
Remote Patient Monitoring: the use of connected electronic tools to record medical data in one location for review by a provider remotely
Mobile Health: health care and public health information provided through mobile devices
General educational information
Targeted texts
Notifications about disease outbreaks
https://www.healthit.gov/topic/health-it-initiatives/telemedicine-and-telehealth
https://chironhealth.com/blog/telemedicine-vs-telehealth-whats-the-difference/
5

## Slide 6
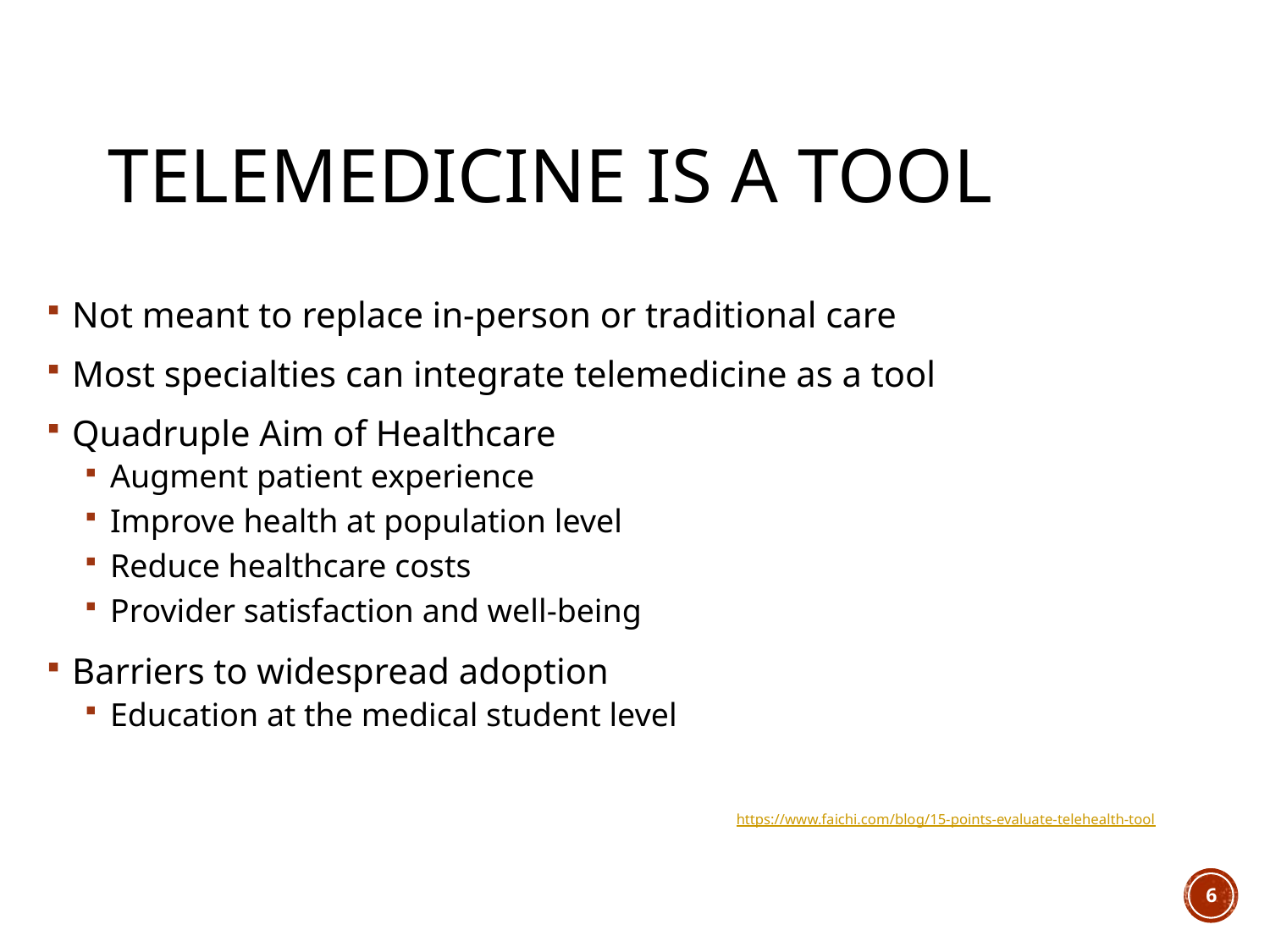

# Telemedicine is a tool
Not meant to replace in-person or traditional care
Most specialties can integrate telemedicine as a tool
Quadruple Aim of Healthcare
Augment patient experience
Improve health at population level
Reduce healthcare costs
Provider satisfaction and well-being
Barriers to widespread adoption
Education at the medical student level
https://www.faichi.com/blog/15-points-evaluate-telehealth-tool
6

## Slide 7
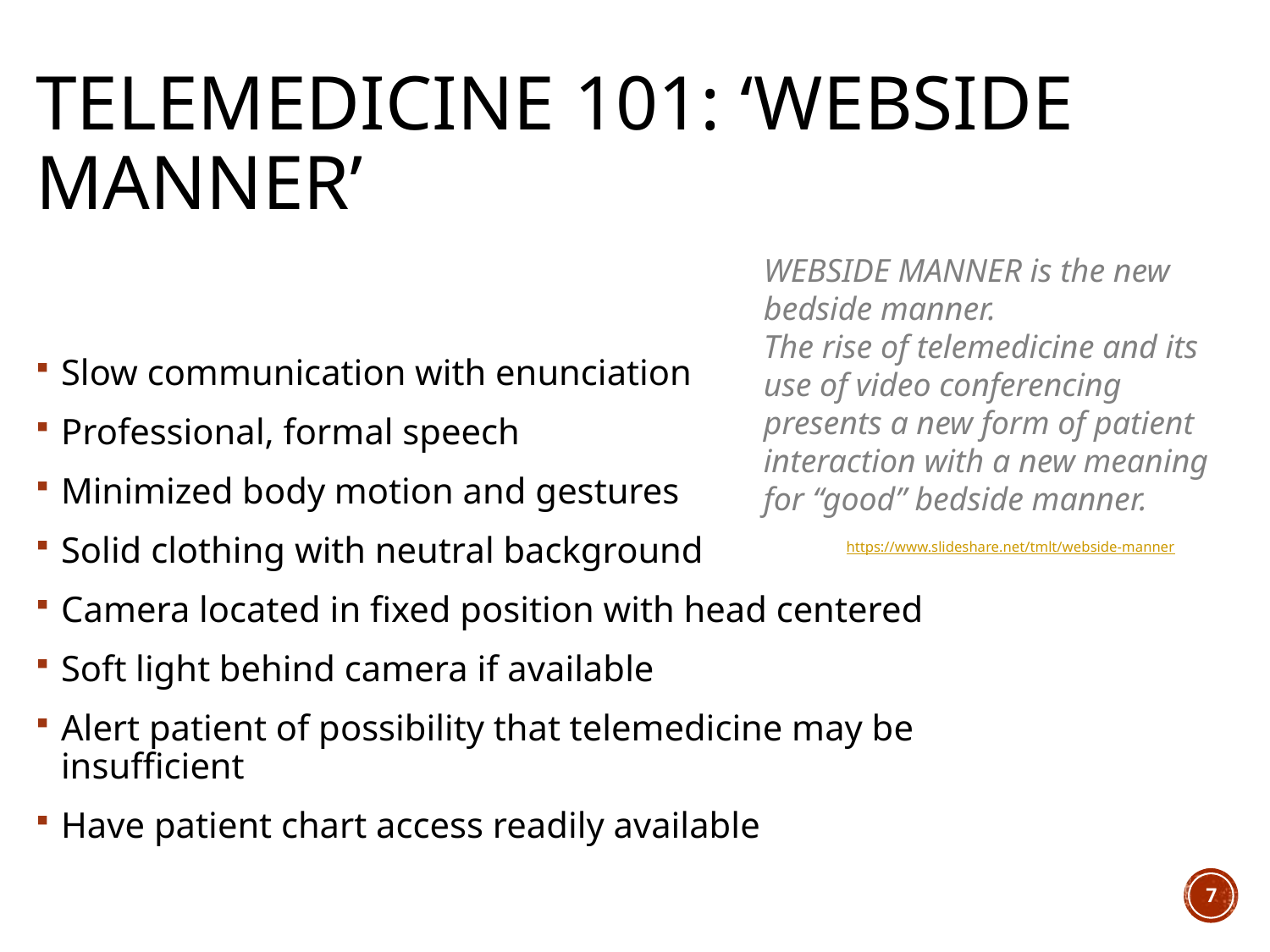

# Telemedicine 101: ‘Webside Manner’
WEBSIDE MANNER is the new bedside manner.
The rise of telemedicine and its use of video conferencing presents a new form of patient interaction with a new meaning for “good” bedside manner.
Slow communication with enunciation
Professional, formal speech
Minimized body motion and gestures
Solid clothing with neutral background
Camera located in fixed position with head centered
Soft light behind camera if available
Alert patient of possibility that telemedicine may be insufficient
Have patient chart access readily available
https://www.slideshare.net/tmlt/webside-manner
7

## Slide 8
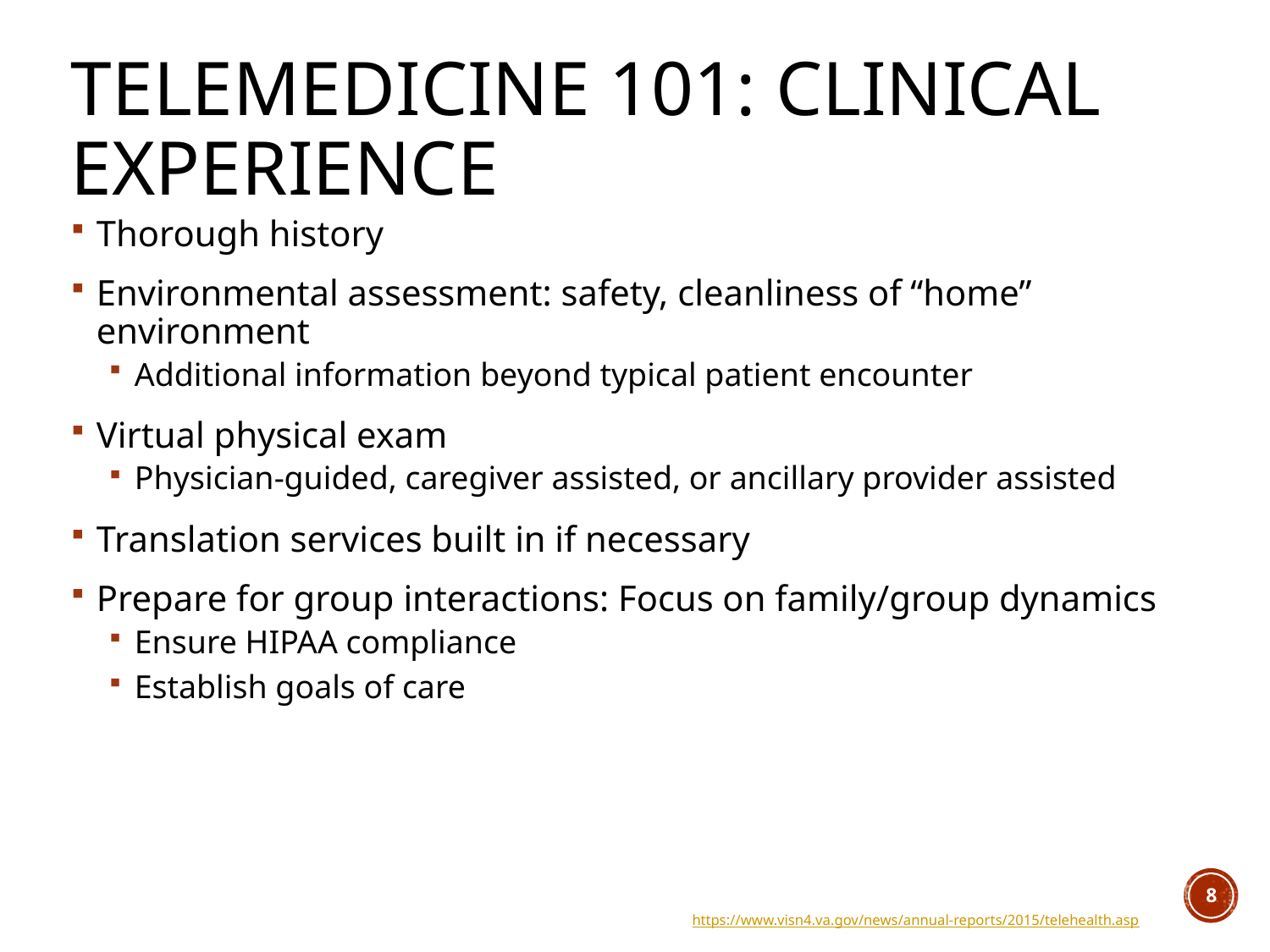

# Telemedicine 101: Clinical Experience
Thorough history
Environmental assessment: safety, cleanliness of “home” environment
Additional information beyond typical patient encounter
Virtual physical exam
Physician-guided, caregiver assisted, or ancillary provider assisted
Translation services built in if necessary
Prepare for group interactions: Focus on family/group dynamics
Ensure HIPAA compliance
Establish goals of care
8
https://www.visn4.va.gov/news/annual-reports/2015/telehealth.asp

## Slide 9
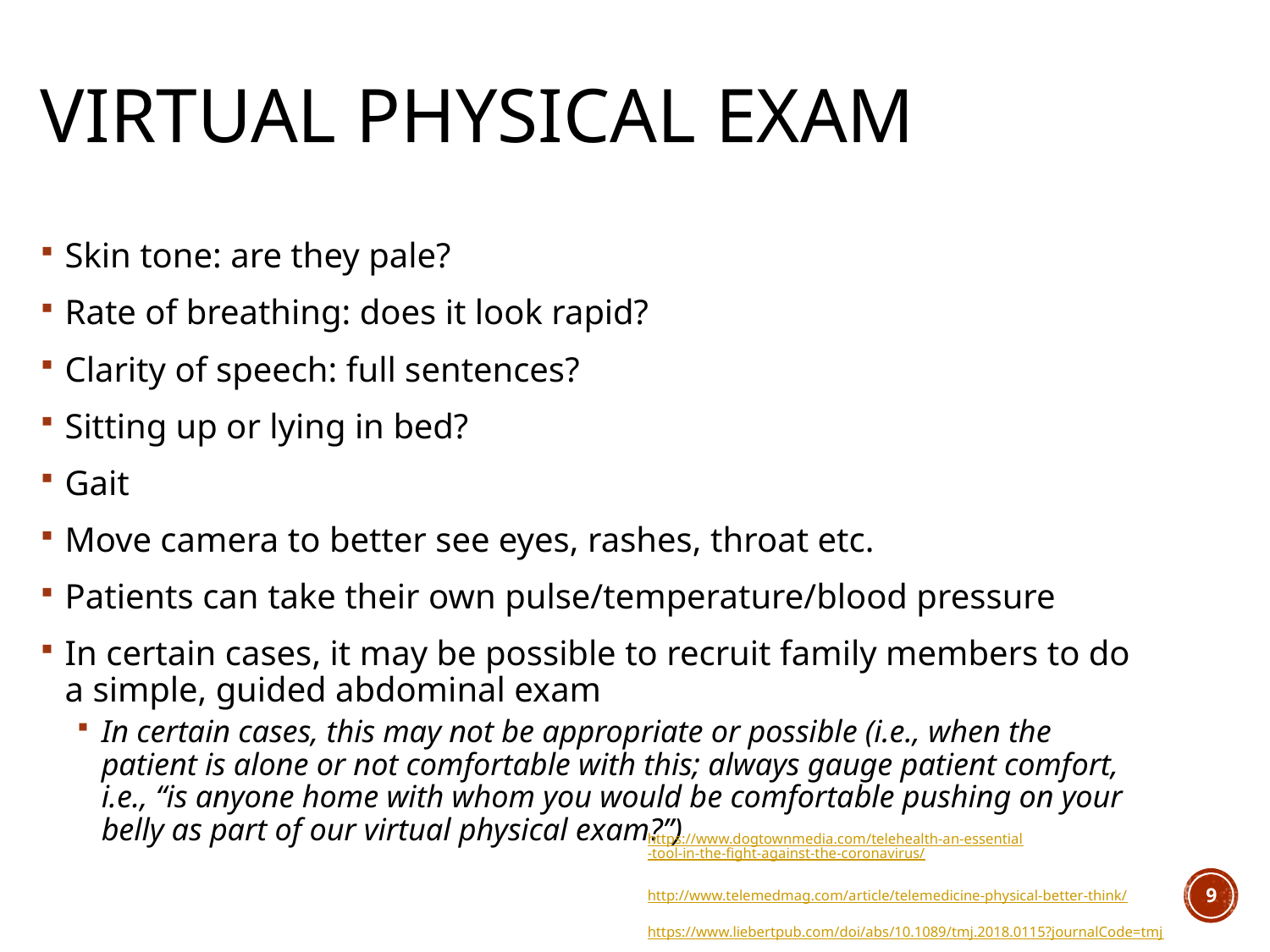

# Virtual Physical Exam
Skin tone: are they pale?
Rate of breathing: does it look rapid?
Clarity of speech: full sentences?
Sitting up or lying in bed?
Gait
Move camera to better see eyes, rashes, throat etc.
Patients can take their own pulse/temperature/blood pressure
In certain cases, it may be possible to recruit family members to do a simple, guided abdominal exam
In certain cases, this may not be appropriate or possible (i.e., when the patient is alone or not comfortable with this; always gauge patient comfort, i.e., “is anyone home with whom you would be comfortable pushing on your belly as part of our virtual physical exam?”)
https://www.dogtownmedia.com/telehealth-an-essential-tool-in-the-fight-against-the-coronavirus/
9
http://www.telemedmag.com/article/telemedicine-physical-better-think/
https://www.liebertpub.com/doi/abs/10.1089/tmj.2018.0115?journalCode=tmj

## Slide 10
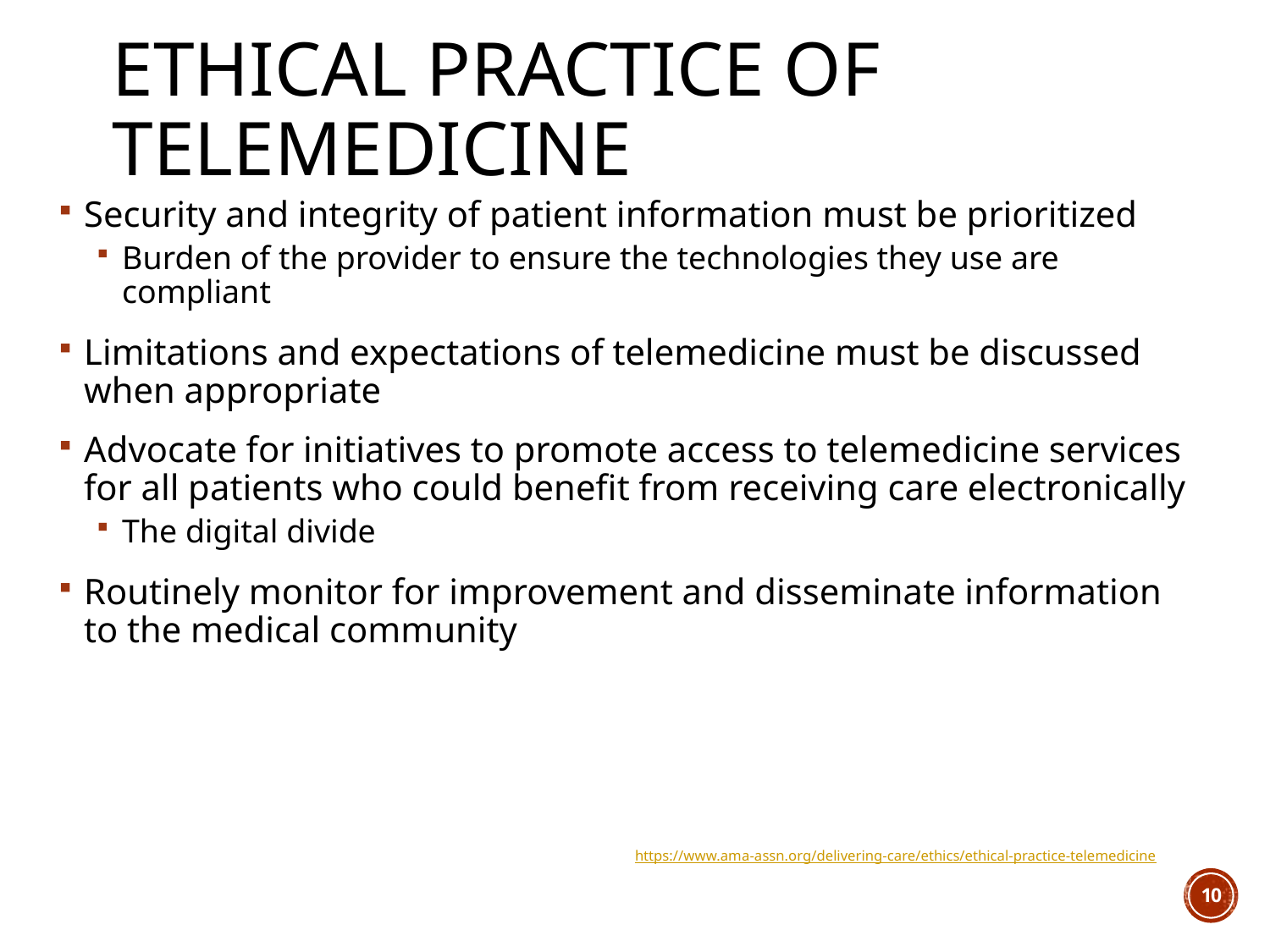

# Ethical Practice of Telemedicine
Security and integrity of patient information must be prioritized
Burden of the provider to ensure the technologies they use are compliant
Limitations and expectations of telemedicine must be discussed when appropriate
Advocate for initiatives to promote access to telemedicine services for all patients who could benefit from receiving care electronically
The digital divide
Routinely monitor for improvement and disseminate information to the medical community
https://www.ama-assn.org/delivering-care/ethics/ethical-practice-telemedicine
10

## Slide 11
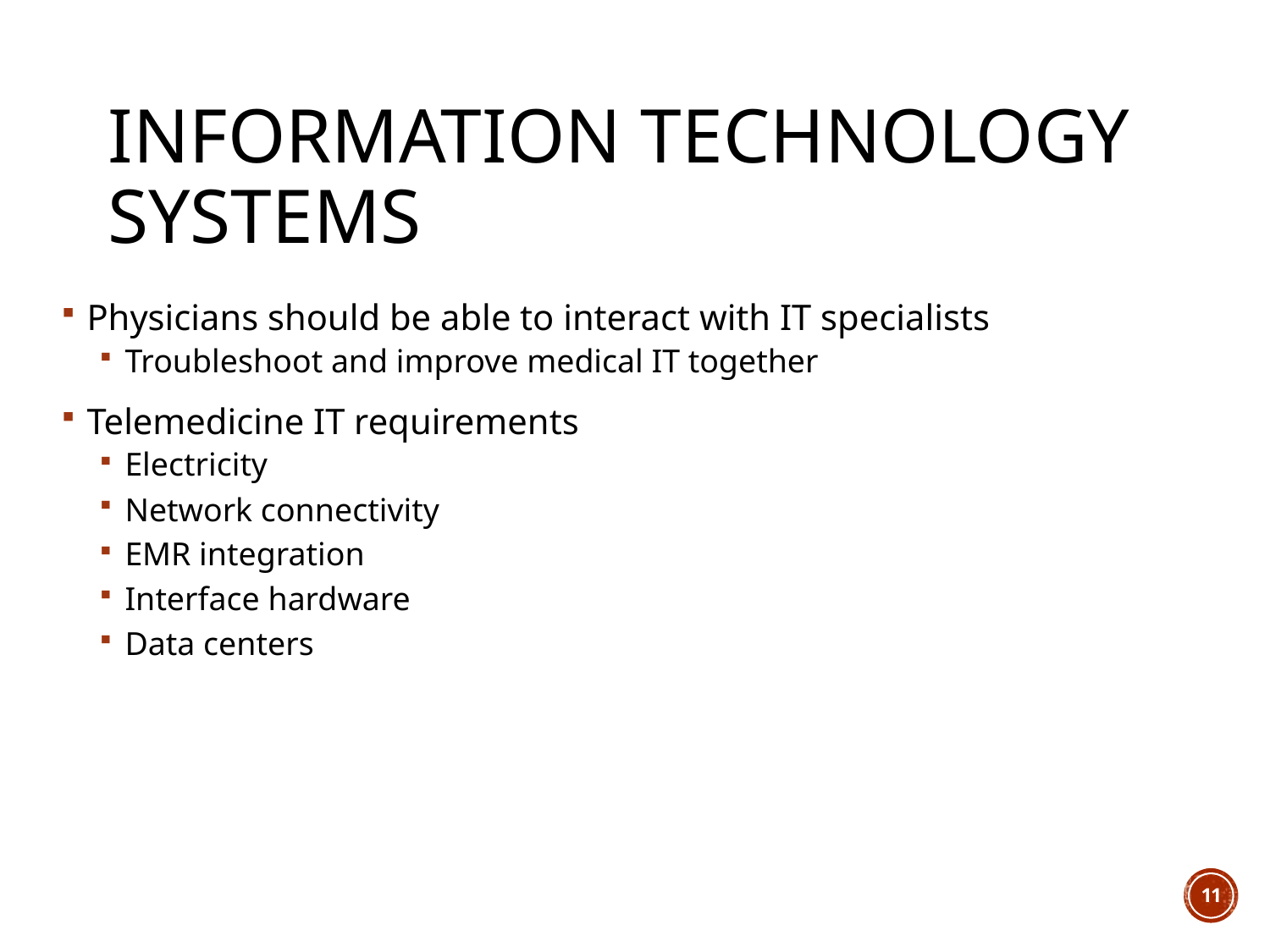

# Information Technology Systems
Physicians should be able to interact with IT specialists
Troubleshoot and improve medical IT together
Telemedicine IT requirements
Electricity
Network connectivity
EMR integration
Interface hardware
Data centers
11

## Slide 12
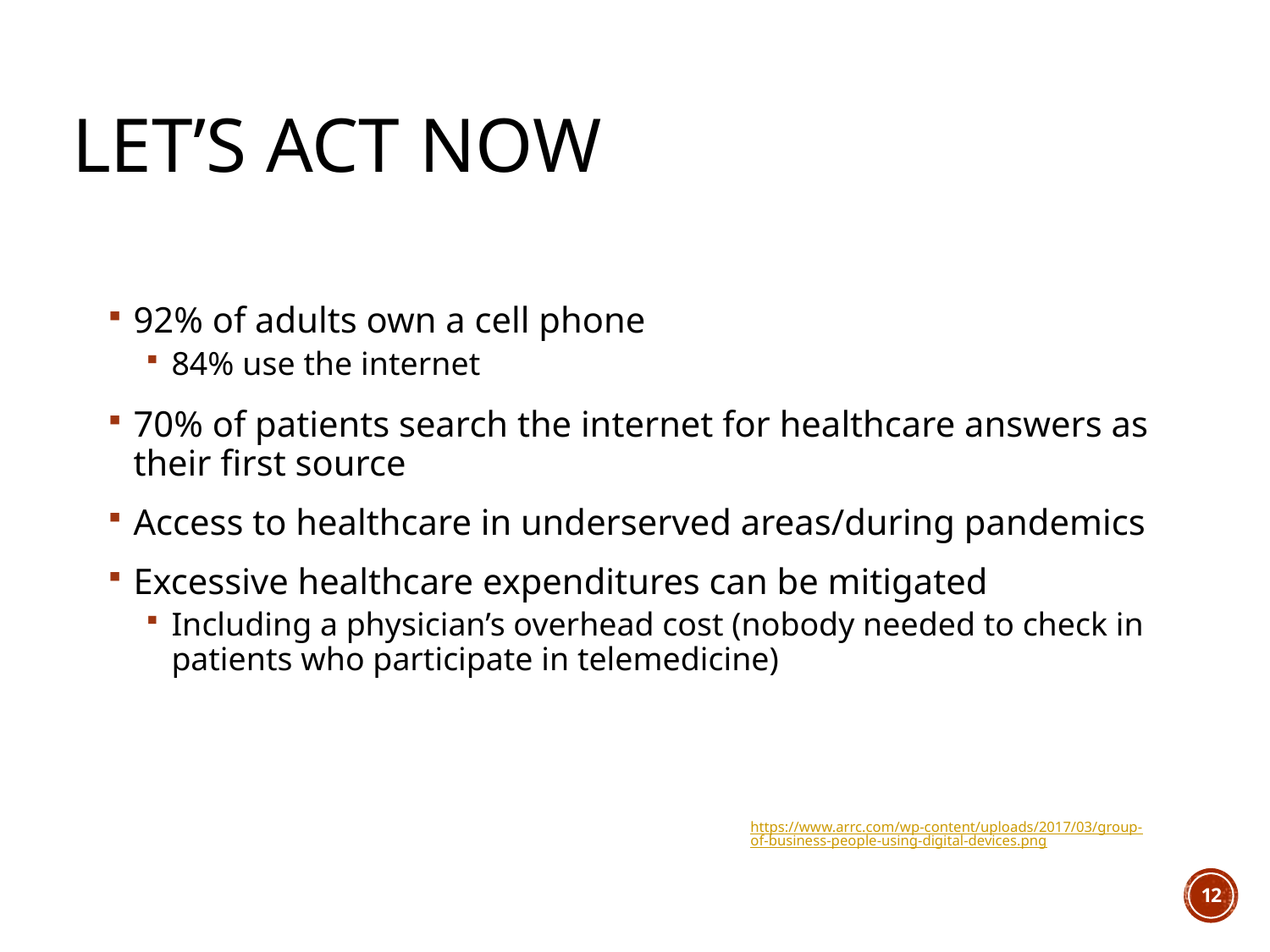

# Let’s Act now
92% of adults own a cell phone
84% use the internet
70% of patients search the internet for healthcare answers as their first source
Access to healthcare in underserved areas/during pandemics
Excessive healthcare expenditures can be mitigated
Including a physician’s overhead cost (nobody needed to check in patients who participate in telemedicine)
https://www.arrc.com/wp-content/uploads/2017/03/group-of-business-people-using-digital-devices.png
12

## Slide 13
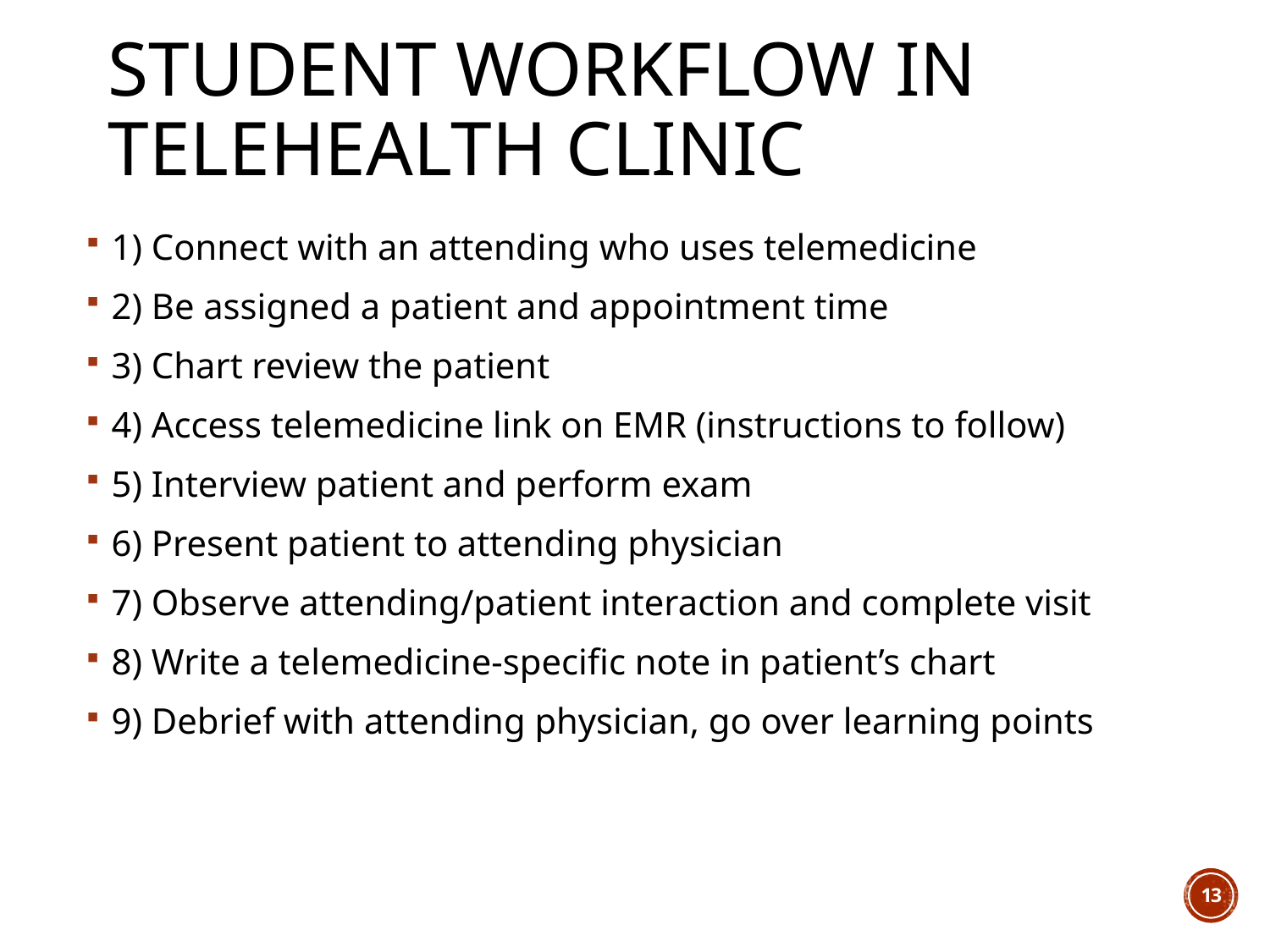

# Student workflow In Telehealth Clinic
1) Connect with an attending who uses telemedicine
2) Be assigned a patient and appointment time
3) Chart review the patient
4) Access telemedicine link on EMR (instructions to follow)
5) Interview patient and perform exam
6) Present patient to attending physician
7) Observe attending/patient interaction and complete visit
8) Write a telemedicine-specific note in patient’s chart
9) Debrief with attending physician, go over learning points
13

## Slide 14
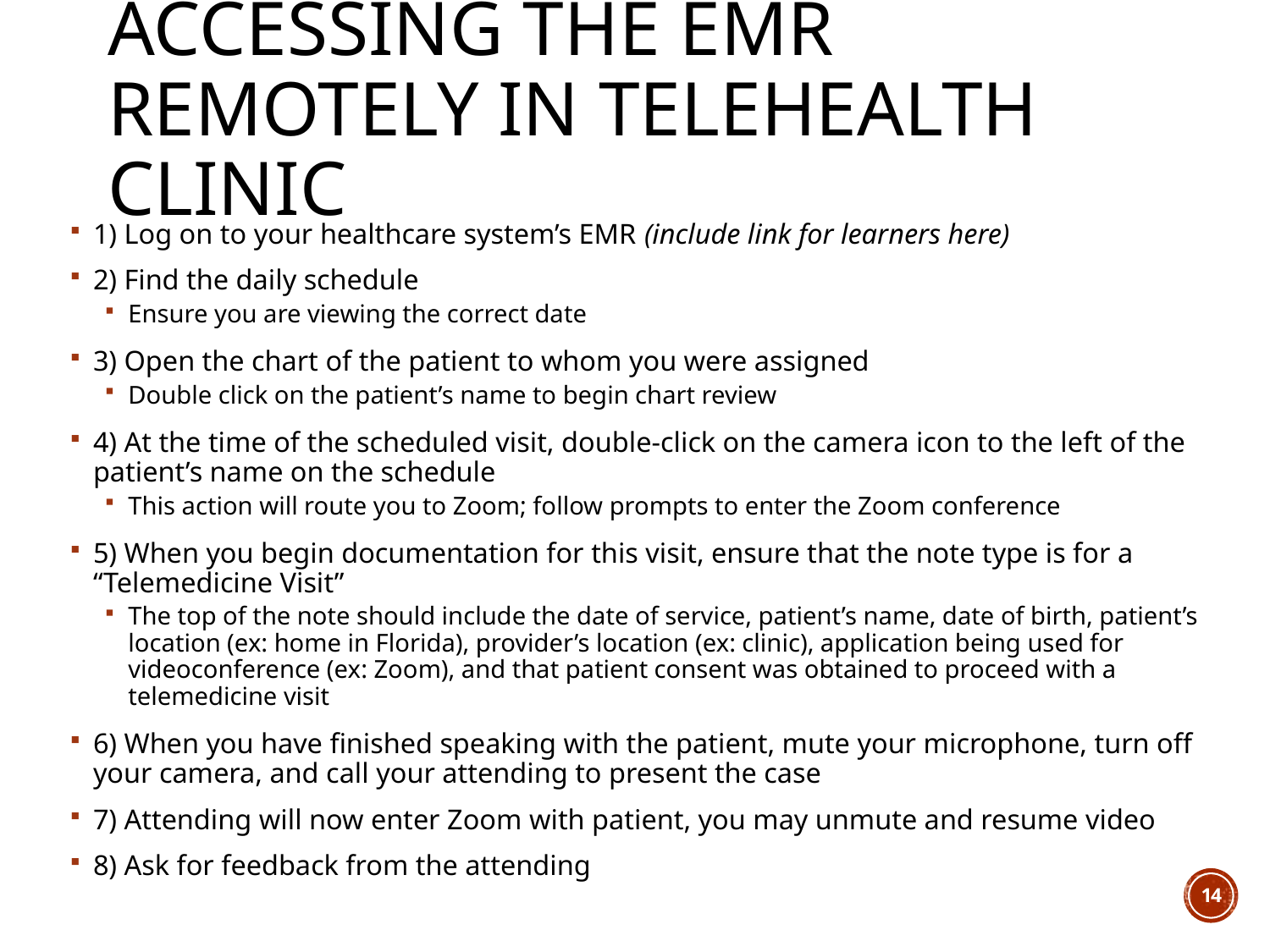

# Accessing the EMR Remotely in Telehealth Clinic
1) Log on to your healthcare system’s EMR (include link for learners here)
2) Find the daily schedule
Ensure you are viewing the correct date
3) Open the chart of the patient to whom you were assigned
Double click on the patient’s name to begin chart review
4) At the time of the scheduled visit, double-click on the camera icon to the left of the patient’s name on the schedule
This action will route you to Zoom; follow prompts to enter the Zoom conference
5) When you begin documentation for this visit, ensure that the note type is for a “Telemedicine Visit”
The top of the note should include the date of service, patient’s name, date of birth, patient’s location (ex: home in Florida), provider’s location (ex: clinic), application being used for videoconference (ex: Zoom), and that patient consent was obtained to proceed with a telemedicine visit
6) When you have finished speaking with the patient, mute your microphone, turn off your camera, and call your attending to present the case
7) Attending will now enter Zoom with patient, you may unmute and resume video
8) Ask for feedback from the attending
14

## Slide 15
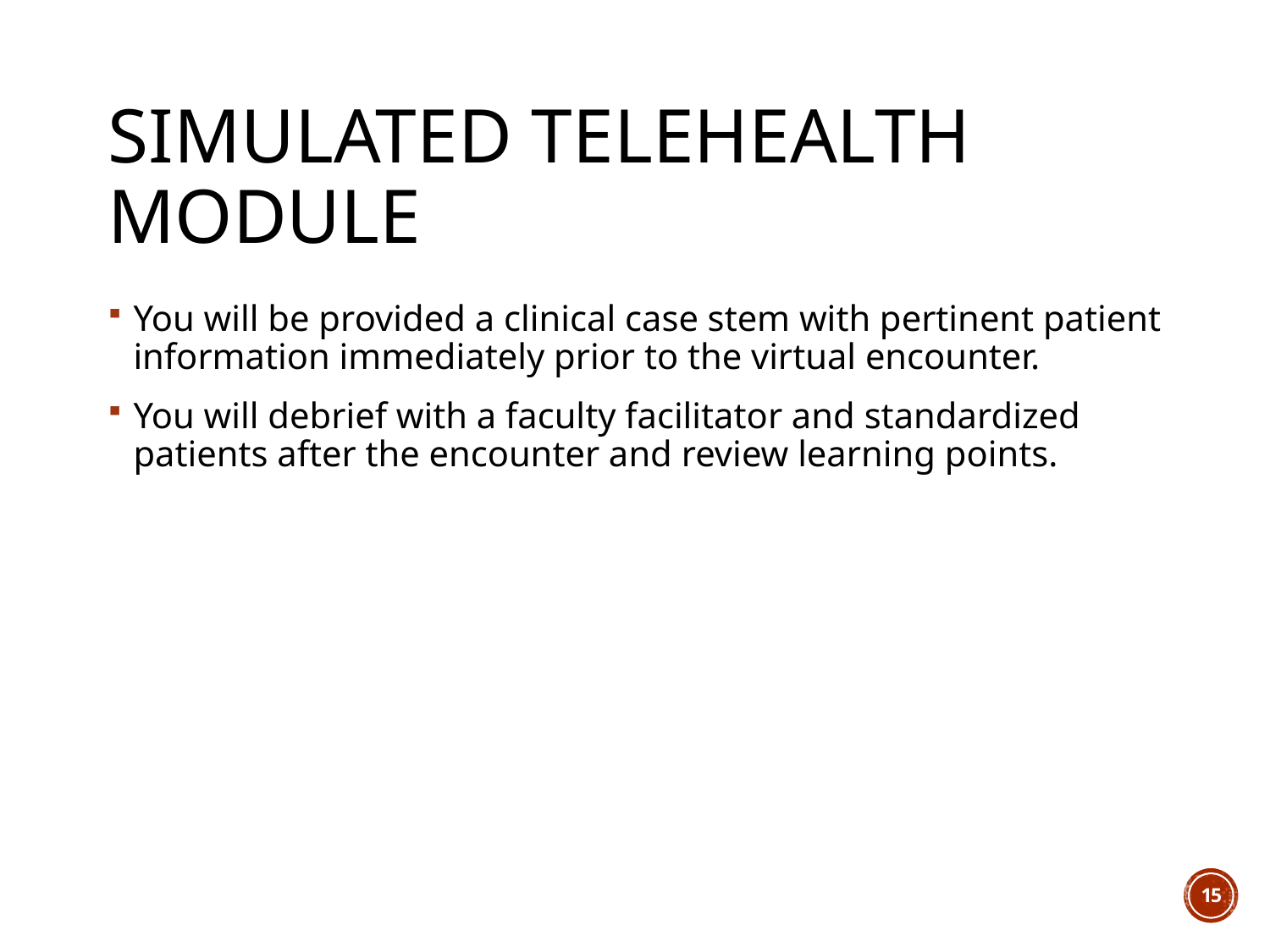

# Simulated Telehealth Module
You will be provided a clinical case stem with pertinent patient information immediately prior to the virtual encounter.
You will debrief with a faculty facilitator and standardized patients after the encounter and review learning points.
15

## Slide 16
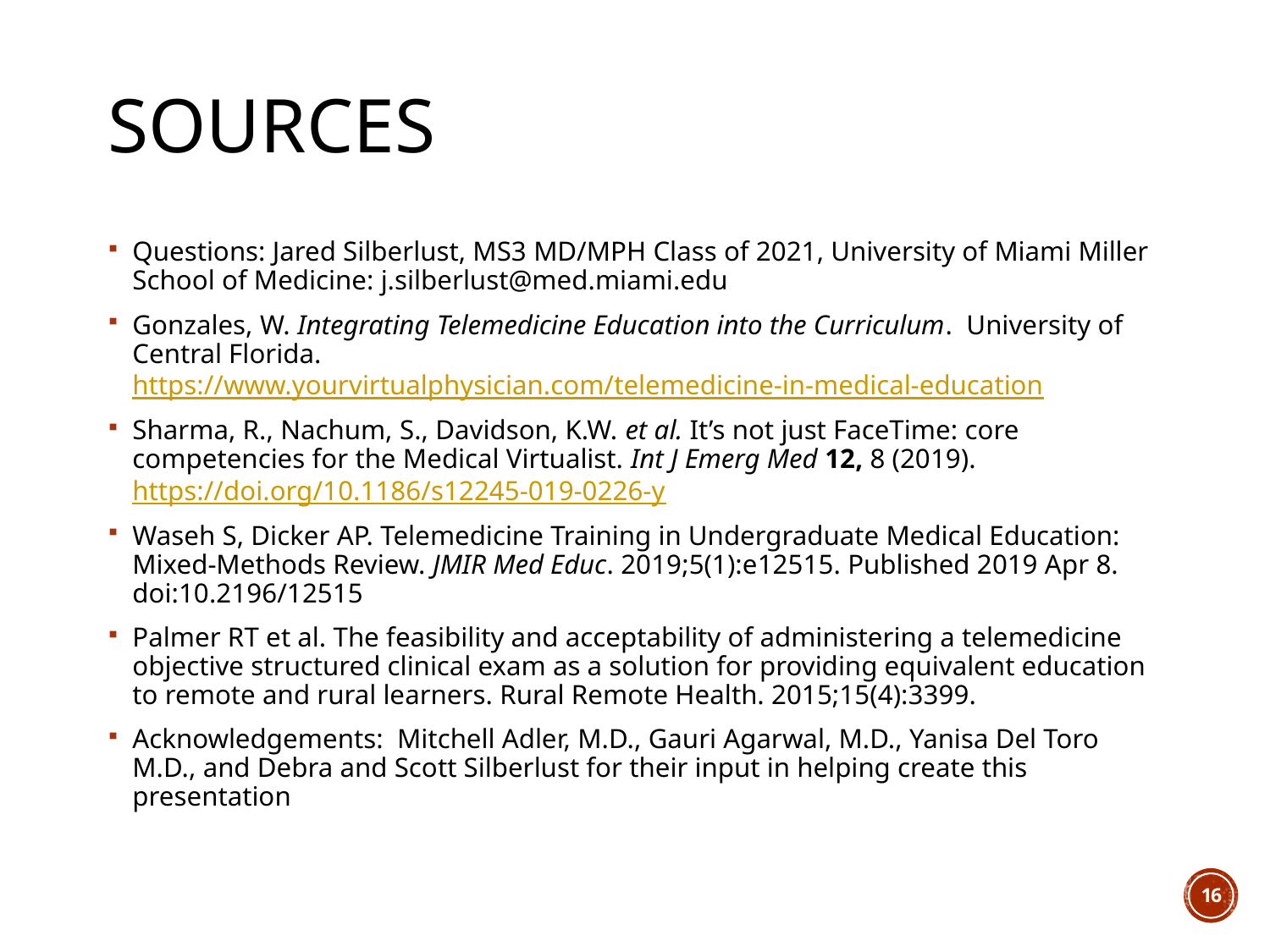

# Sources
Questions: Jared Silberlust, MS3 MD/MPH Class of 2021, University of Miami Miller School of Medicine: j.silberlust@med.miami.edu
Gonzales, W. Integrating Telemedicine Education into the Curriculum. University of Central Florida. https://www.yourvirtualphysician.com/telemedicine-in-medical-education
Sharma, R., Nachum, S., Davidson, K.W. et al. It’s not just FaceTime: core competencies for the Medical Virtualist. Int J Emerg Med 12, 8 (2019). https://doi.org/10.1186/s12245-019-0226-y
Waseh S, Dicker AP. Telemedicine Training in Undergraduate Medical Education: Mixed-Methods Review. JMIR Med Educ. 2019;5(1):e12515. Published 2019 Apr 8. doi:10.2196/12515
Palmer RT et al. The feasibility and acceptability of administering a telemedicine objective structured clinical exam as a solution for providing equivalent education to remote and rural learners. Rural Remote Health. 2015;15(4):3399.
Acknowledgements: Mitchell Adler, M.D., Gauri Agarwal, M.D., Yanisa Del Toro M.D., and Debra and Scott Silberlust for their input in helping create this presentation
16
