# Supplementary material for: Situational Awareness in Telehealth: A Virtual Standardized Patient Case for Transitioning Preclinical to Clinical Medical Students
Source: MedEdPORTAL. 2025 Apr 11;21:11517. doi: 10.15766/mep_2374-8265.11517 (PMC11985545; doi:10.15766/mep_2374-8265.11517)
Supplement: Supplementary file 1 — Student Prework.pptxFaculty Training Guide.docxSP Scenario.docxSP Survey Tool.docxScenario Stem.pptxStudent Prebriefing.pptxSession Facilitators Presentation.pptxPostencounter Student Survey.docx [file mep_2374-8265.11517-s001.zip › B. Faculty Training Guide.docx]

**UM-JMH Center for Patient Safety – Faculty Training Guide**

**Transition to Clerkships Bootcamp**

**Telehealth Patient Encounter**

**Telehealth Patient Encounter**


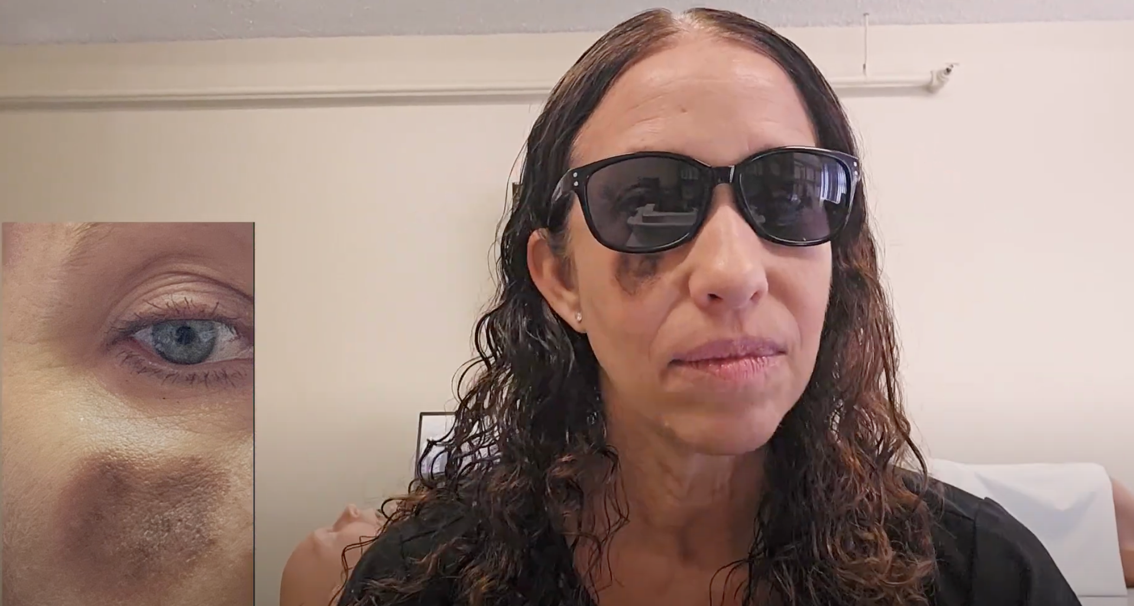


*Author-owned image*

**Description:**

Participating in this year’s course are approximately 150 MD and 50 MD/MPH pre-clinical medical students. The students have had limited clinical experience to date. The telehealth module is designed for the students to obtain an initial focused history exam. They will be asked to consult a patient with the chief complaint of palpitations for the past several months that have progressed in severity. Upon entering the breakout room, they will notice the standardized patient wearing dark sunglasses and has an apparent skin lesion that appears slightly below the rim of the glasses. To maintain psychological safety, we inform the students that the sessions are not recorded.

This scenario provides opportunities to discuss:

- How patient centric contextual clues may be helpful in forming a differential diagnosis, especially with a patient who is a poor historian or withholding information.
- The importance of conducting focused and coordinated history taking in a timely and patient-sensitive manner.
- The importance of clear, concise communication with patients during telemedicine encounters.
- Overcoming challenges of establishing rapport in a telehealth setting.
- Overcoming challenges of social relational barriers that may limit full exploration of a patient’s health status.
- The importance of situational awareness.

**Set-up description:**

Two (2) Zoom sessions will be run in parallel. Each virtually held session will have 4 breakout rooms which include 3 breakout rooms for the telehealth encounter and one breakout room for the debriefing. A facilitator will be responsible for timing the rooms as well as moving the students, faculty and SPs to the breakout rooms.

*Please refer to the flow chart for detailed information.

**Timing:**

Pre-briefing: 10-minutes

Telehealth Encounter: 8-minutes

SP Evaluation: 3-minutes

Debriefing: 15-minutes

**Objectives:**

By the end of this activity participants should be able to:

1. Outline key components of a typical telehealth encounter.
2. List effective strategies to reduce communication errors in the telehealth clinical environment.
3. Demonstrate a focused patient assessment, including assurance of proper patient, setting, and confidentiality during a telehealth visit.
4. Explain the vital components of active situational awareness (SA) in the telehealth clinical environment.

**Instructions to Learners:**

This activity is designed for you to gain Telehealth experience.  You will not be graded, and the sessions are not being recorded.  This encounter is for your learning experience only.​

- Please meet with Melanie / Michael Jones who presents for a Telehealth visit. The patient has had palpitations for the last several months which have progressed in severity. The patient had an episode last night and decided to request this appointment.​
- Please take a focused history.  You will have 8 minutes to complete the patient encounter.  A message will be displayed when you have 2 minutes remaining and again when your time is up.  ​
- There may be a faculty member observing in your session (with camera off).​
- Once the Telehealth encounter is completed, you will receive a notice to move to the debriefing room.  There, you may wait up to 11 minutes while the rest of your group completes the encounter.  After which, you will be joined by the rest of your group, a faculty member, and eventually the standardized patients.  ​
- During the debriefing, please use the opportunity to reflect on what went well and what could have been done differently.    ​
- At the conclusion of the 15-minute debriefing, please ‘Leave the Meeting.’

**Standardized Patient (SP) instructions: (Melanie Jones or Matthew Jones)**

Approximately 10 SPs were trained to allow for flexibility in scheduling.  The SPs are hires from the UHealth Standardized Participant Program. They were provided with the case details and attended a one-hour training session in preparation for this module. You have been provided a copy of the SP guide which includes all case details and guidance for how to answer certain questions to maintain continuity between encounters (see Simulated Patient Scenario, appendix C).

Following the simulation session, you will debrief with the students. See the following pages for debriefing guidelines.

Faculty debriefing guide:

- 15-minute debriefing
- The SPs have been informed to keep their sunglasses on during the debrief until you ask that they remove them.

After introducing yourself, please establish psychological safety with the learners. Remind them of the following:

This activity is designed to help you gain telehealth experience.  You are not being graded, and the sessions are not being recorded.  This encounter is for your learning experience only. This is a safe space. We ask that you speak openly, respect one another, and maintain the confidentiality of this space and of your colleagues.

**Suggested questions you may ask the students:**

- How do you think the encounter went?
- What went well? What would you have done differently?
- What was your initial reaction when seeing the patient?
- How did you confirm that you were speaking to the correct patient?
- What actions did you take to establish rapport via a telehealth mode of interaction?
  - Was it a challenge for anyone?
- How was the flow of information?
  - Was communication clear?
  - Was closed loop communication used?
  - Were you comfortable asking open-ended questions to gain information?
- You had about 8 minutes to meet with the patient. What was your strategy for a focused patient exam given the short timeframe?
- Now that you’ve experienced a telehealth encounter, what are some key elements you would instill when using a virtual platform for a patient encounter?
  - For example, how important is your Zoom background, a quiet area, well-lit room, being well-framed into the camera, and your facial expression?

We have established the importance of creating an environment that allows you to build rapport with the patient. Now, let’s talk about cues specific to the patient that you may have noticed.

With only a show only of hands, and please be honest, how many of you asked your patient to remove his/her sunglasses?

- For those who did not ask the SP to remove his/her glasses, can you tell me why you didn’t?

*(Afterwards, please ask the SPs to remove their sunglasses*.)

As you’ll see, this patient was wearing sunglasses to cover an embarrassing facial lesion that may or may not be skin cancer. The simulated patients were prepared to answer questions regarding the lesion if asked.

For this particular case, the patient used sunglasses to cover a facial lesion. It is important to keep in mind that another patient may be hiding a bruise that is attributed to an abusive relationship. It is your responsibility to ask your patient questions regarding personal safety and harm risk assessment.

- Outside of the clinical environment, it may feel unnatural to ask a patient about a lesion or to ask them to remove their glasses; but hopefully, this experience has helped shed light on the importance of being aware of subtle cues during patient encounters (both via telehealth and in person), and putting aside societal norms to investigate these cues for the sake of patient care.
- Who here has heard of the term “situational awareness?” (show of hands); can any of you define the term?
  - SA is the perception of elements of the environment within a volume of time and space, the comprehension of their meaning and the projection of their status into the near future
  - There are 3 levels of SA:
    - Level 1 involves perceiving environmental cues and collecting diagnostic information.
      - Example from scenario: noticing the patient is wearing sunglasses during the visit (outside the norm), during the telehealth visit, and indoors.
    - Level 2 requires comprehending this information to assess the situation accurately.
      - Example from scenario: asking questions pertaining to reason for sunglasses regarding differential diagnosis (vision problems, dizziness, covering for something (lesion/bruise))
    - Level 3 involves planning clinical outcomes by predicting future events for effective treatment.
      - Example from scenario: make suggestions to pursue further (come in for an in person visit to analyze, biopsy lesion, assess for danger or harm, assess for current safety)
- What are strategies to broaching uncomfortable situations/topics with the patient?
  - Discuss Advocacy/Inquiry
  - Examples that Faculty debriefer may consider mentioning:
    - Acknowledge potential discomfort: “I understand you may feel more comfortable wearing your sunglasses, but do you mind removing them for one moment to allow me to complete my physical assessment?”
    - Remind patient of role: “As your physician, it is my responsibility to look after your overall wellbeing. I notice what might be a lesion or bruise under your sunglasses, can you remove them for a moment for me to examine you more closely?”
    - Acknowledge telehealth limitations: “Of course, we can only do so much in this format, but I need to be able to visualize all that I am able while we are on, so would you mind removing your sunglasses (increasing the light, turning down the background volume, etc.)”
- How do we bridge to the next step if something is concerning (i.e., how do we suggest that the patient return for an in-person visit)?
  - Lesion: “I understand you are seeing me today to discuss your palpitations, however, I would like to have you come in person as soon as possible for a visit to take a closer look at the lesion under your eye.”
  - Bruise: If you have concern of abuse but maybe you don’t know if they are truly alone (someone offscreen during the telehealth), you can use the palpitations as a plausible reason to ask the patient to return for an in person visit for further workup, at which time you may also broach safety concerns.

**Request feedback from the SPs on their perspective regarding the interaction.**
