# Supplementary material for: Situational Awareness in Telehealth: A Virtual Standardized Patient Case for Transitioning Preclinical to Clinical Medical Students
Source: MedEdPORTAL. 2025 Apr 11;21:11517. doi: 10.15766/mep_2374-8265.11517 (PMC11985545; doi:10.15766/mep_2374-8265.11517)
Supplement: Supplementary file 1 — Student Prework.pptxFaculty Training Guide.docxSP Scenario.docxSP Survey Tool.docxScenario Stem.pptxStudent Prebriefing.pptxSession Facilitators Presentation.pptxPostencounter Student Survey.docx [file mep_2374-8265.11517-s001.zip › E. Scenario Stem.pptx]

## Slide 1
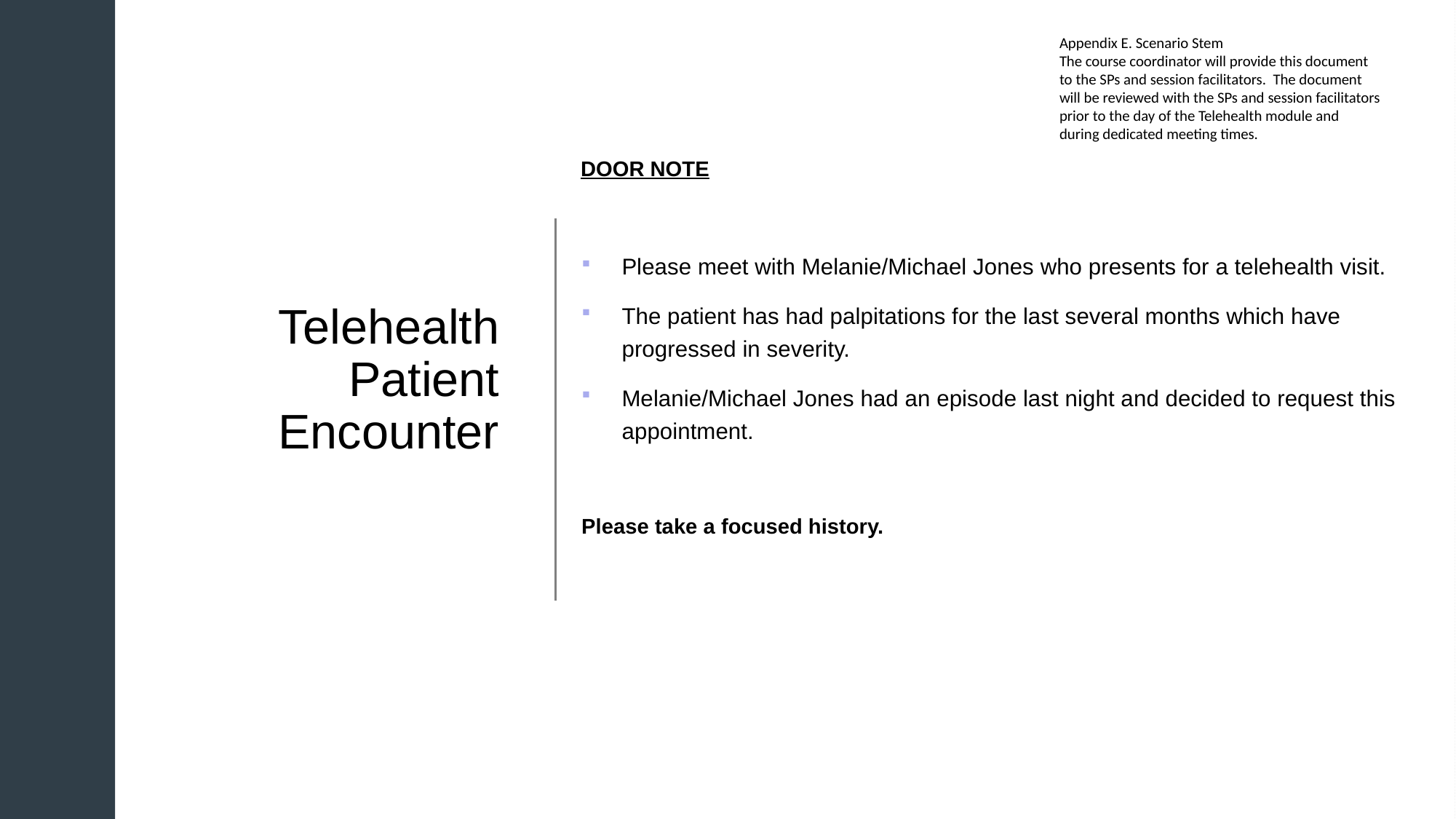

Appendix E. Scenario Stem
The course coordinator will provide this document to the SPs and session facilitators. The document will be reviewed with the SPs and session facilitators prior to the day of the Telehealth module and during dedicated meeting times.
DOOR NOTE
Please meet with Melanie/Michael Jones who presents for a telehealth visit.
The patient has had palpitations for the last several months which have progressed in severity.
Melanie/Michael Jones had an episode last night and decided to request this appointment.
Please take a focused history.
# Telehealth Patient Encounter
