# Supplementary material for: Situational Awareness in Telehealth: A Virtual Standardized Patient Case for Transitioning Preclinical to Clinical Medical Students
Source: MedEdPORTAL. 2025 Apr 11;21:11517. doi: 10.15766/mep_2374-8265.11517 (PMC11985545; doi:10.15766/mep_2374-8265.11517)
Supplement: Supplementary file 1 — Student Prework.pptxFaculty Training Guide.docxSP Scenario.docxSP Survey Tool.docxScenario Stem.pptxStudent Prebriefing.pptxSession Facilitators Presentation.pptxPostencounter Student Survey.docx [file mep_2374-8265.11517-s001.zip › F. Student Prebriefing.pptx]

## Slide 1
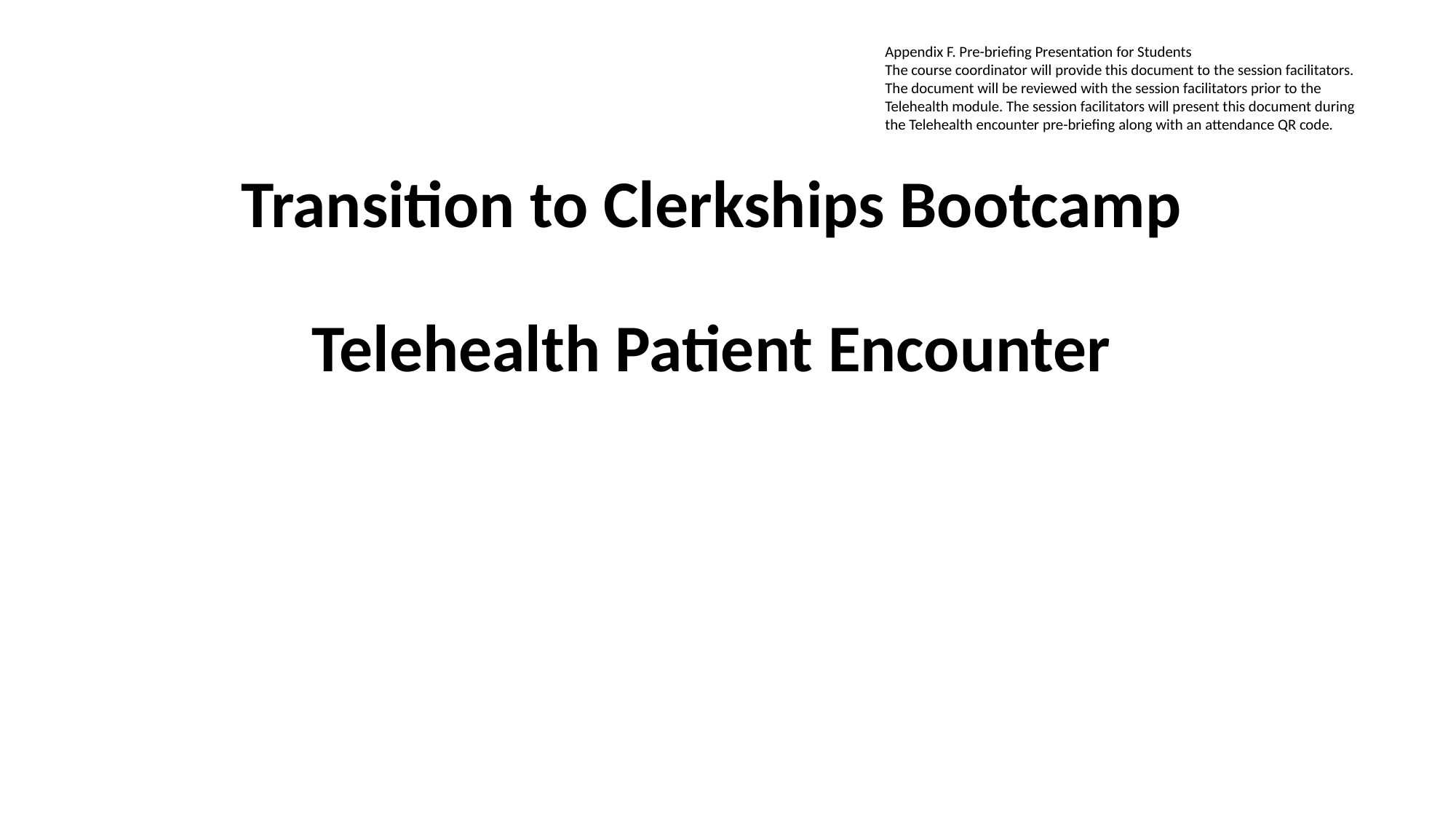

Appendix F. Pre-briefing Presentation for Students
The course coordinator will provide this document to the session facilitators. The document will be reviewed with the session facilitators prior to the Telehealth module. The session facilitators will present this document during the Telehealth encounter pre-briefing along with an attendance QR code.
# Transition to Clerkships Bootcamp Telehealth Patient Encounter

## Slide 2
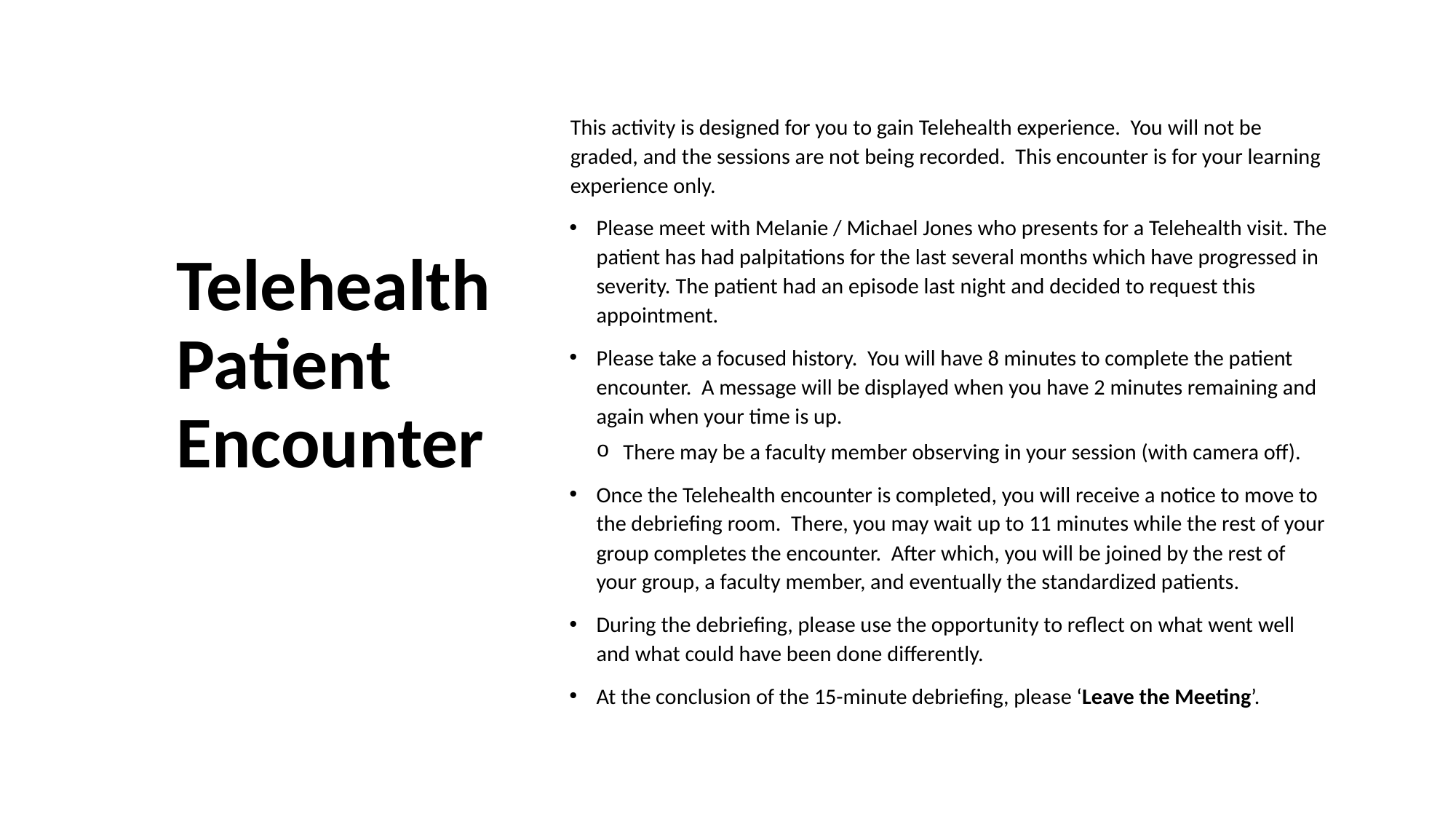

This activity is designed for you to gain Telehealth experience. You will not be graded, and the sessions are not being recorded. This encounter is for your learning experience only.
Please meet with Melanie / Michael Jones who presents for a Telehealth visit. The patient has had palpitations for the last several months which have progressed in severity. The patient had an episode last night and decided to request this appointment.
Please take a focused history. You will have 8 minutes to complete the patient encounter. A message will be displayed when you have 2 minutes remaining and again when your time is up.
There may be a faculty member observing in your session (with camera off).
Once the Telehealth encounter is completed, you will receive a notice to move to the debriefing room. There, you may wait up to 11 minutes while the rest of your group completes the encounter. After which, you will be joined by the rest of your group, a faculty member, and eventually the standardized patients.
During the debriefing, please use the opportunity to reflect on what went well and what could have been done differently.
At the conclusion of the 15-minute debriefing, please ‘Leave the Meeting’.
# Telehealth Patient Encounter
