# Supplementary material for: Situational Awareness in Telehealth: A Virtual Standardized Patient Case for Transitioning Preclinical to Clinical Medical Students
Source: MedEdPORTAL. 2025 Apr 11;21:11517. doi: 10.15766/mep_2374-8265.11517 (PMC11985545; doi:10.15766/mep_2374-8265.11517)
Supplement: Supplementary file 1 — Student Prework.pptxFaculty Training Guide.docxSP Scenario.docxSP Survey Tool.docxScenario Stem.pptxStudent Prebriefing.pptxSession Facilitators Presentation.pptxPostencounter Student Survey.docx [file mep_2374-8265.11517-s001.zip › G. Session Facilitators Presentation.pptx]

## Slide 1
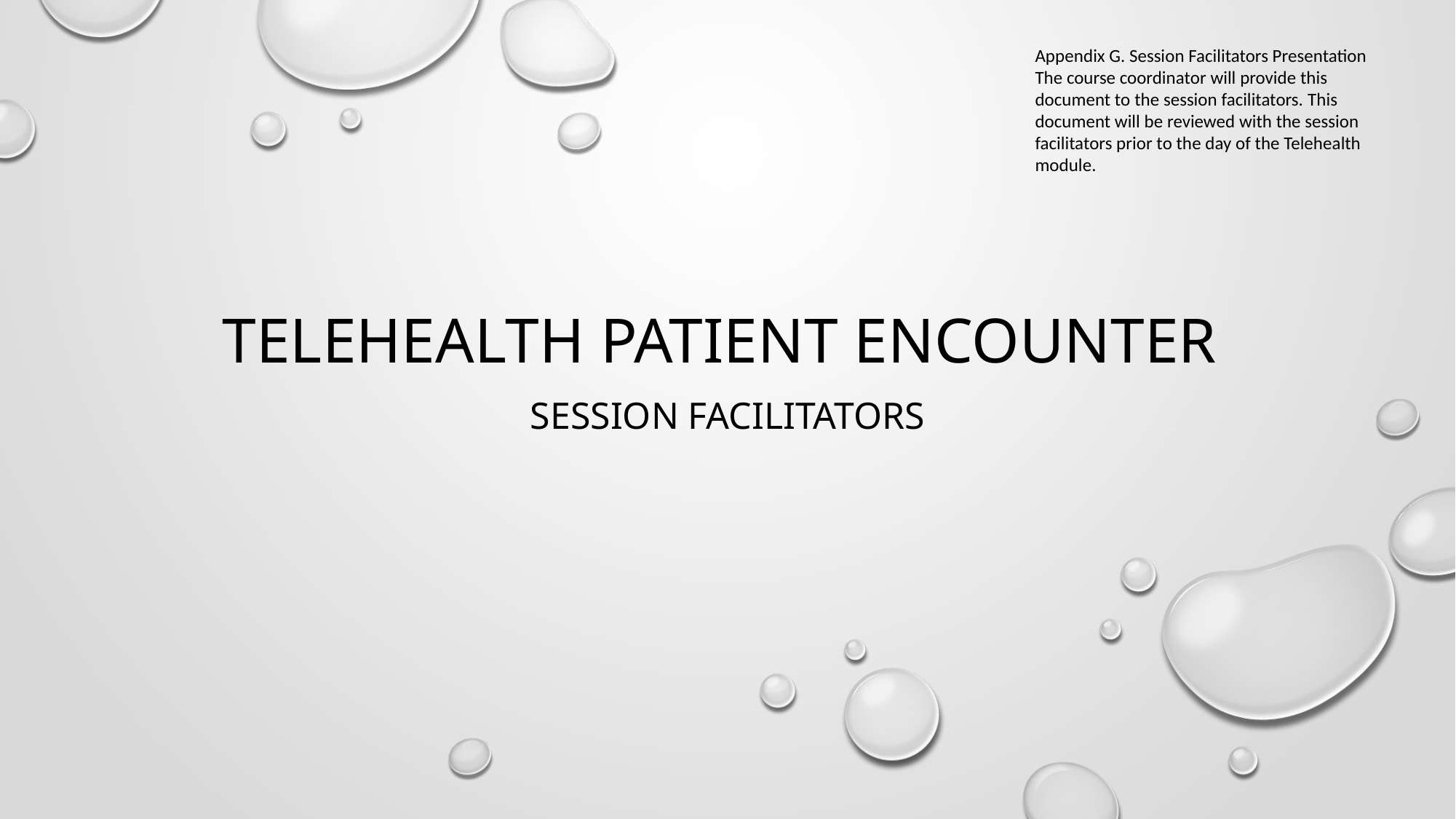

Appendix G. Session Facilitators Presentation
The course coordinator will provide this document to the session facilitators. This document will be reviewed with the session facilitators prior to the day of the Telehealth module.
# Telehealth Patient encounter
SESSION FACILITATORS

## Slide 2
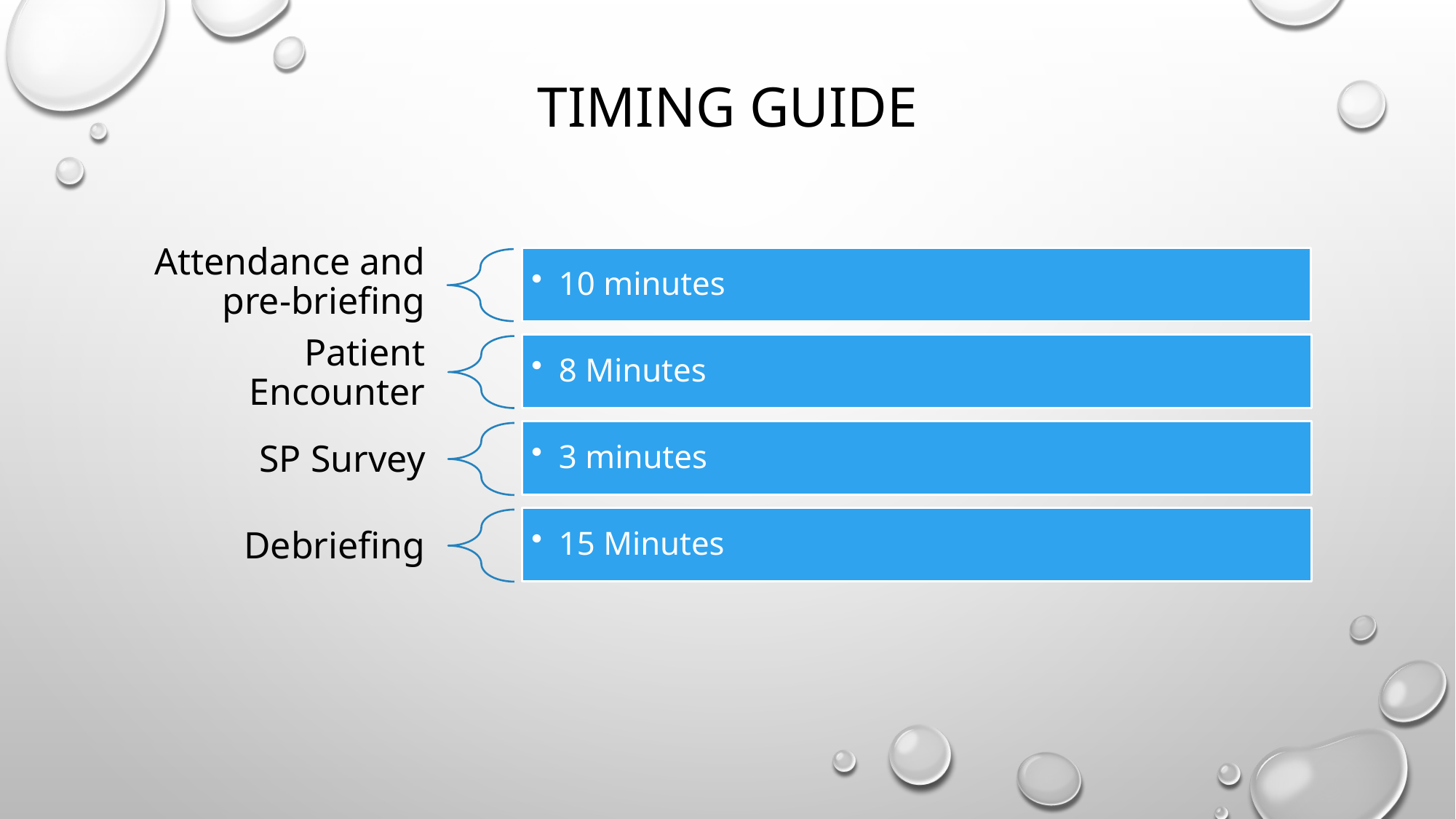

# Timing guide
Attendance and pre-briefing
10 minutes
8 Minutes
Patient Encounter
3 minutes
SP Survey
15 Minutes
Debriefing

## Slide 3
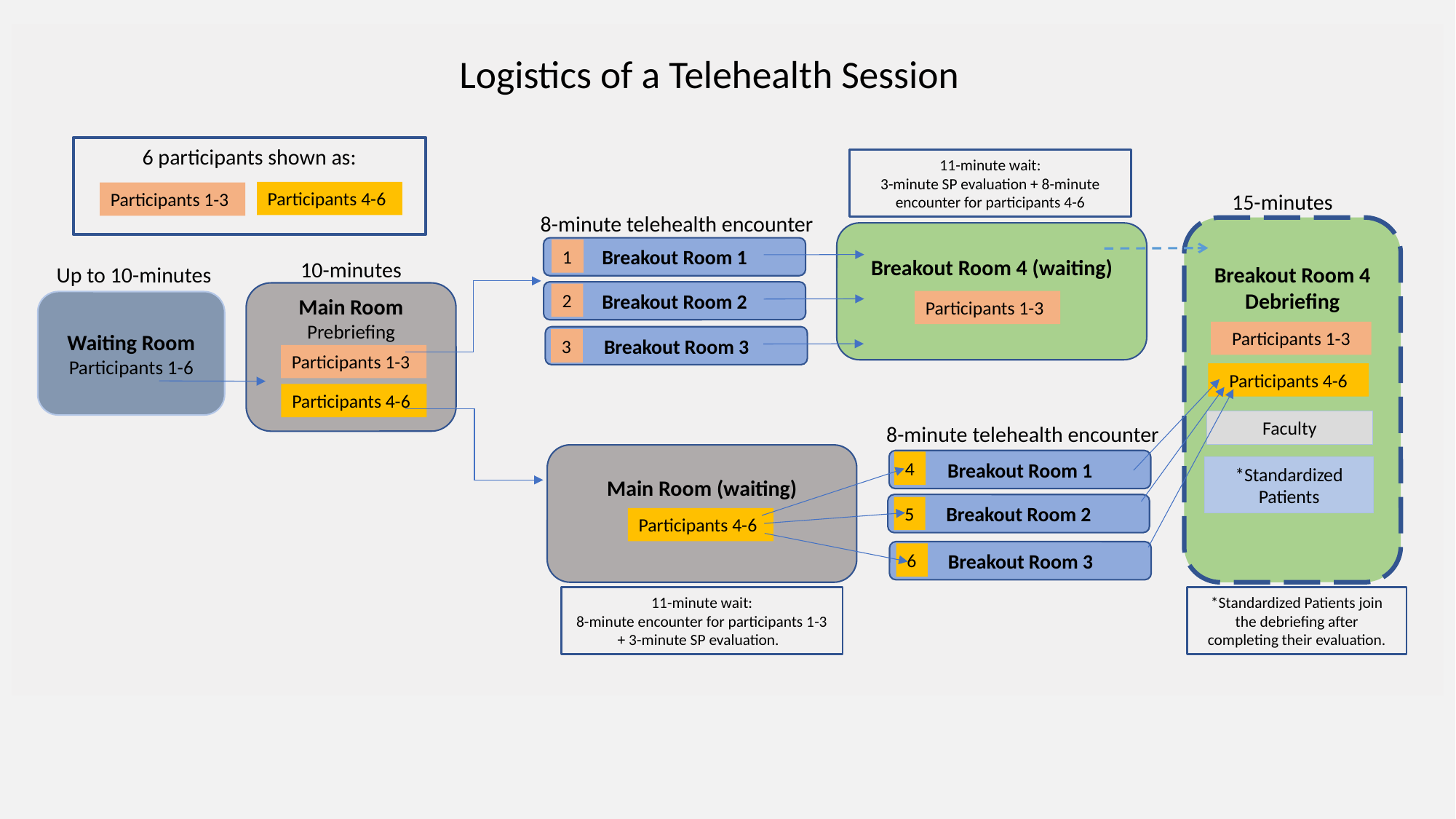

Logistics of a Telehealth Session
6 participants shown as:
11-minute wait:
3-minute SP evaluation + 8-minute encounter for participants 4-6
Participants 4-6
Participants 1-3
15-minutes
8-minute telehealth encounter
Breakout Room 4
Debriefing
Breakout Room 4 (waiting)
Breakout Room 1
1
10-minutes
Up to 10-minutes
Breakout Room 2
Main Room
Prebriefing
2
Participants 1-3
Waiting Room
Participants 1-6
Participants 1-3
Participants 1-3
Breakout Room 3
3
Participants 1-3
Participants 4-6
Participants 4-6
Faculty
8-minute telehealth encounter
Main Room (waiting)
Breakout Room 1
4
*Standardized Patients
Breakout Room 2
5
Participants 4-6
Breakout Room 3
6
11-minute wait:
8-minute encounter for participants 1-3
 + 3-minute SP evaluation.
*Standardized Patients join the debriefing after completing their evaluation.
#

## Slide 4
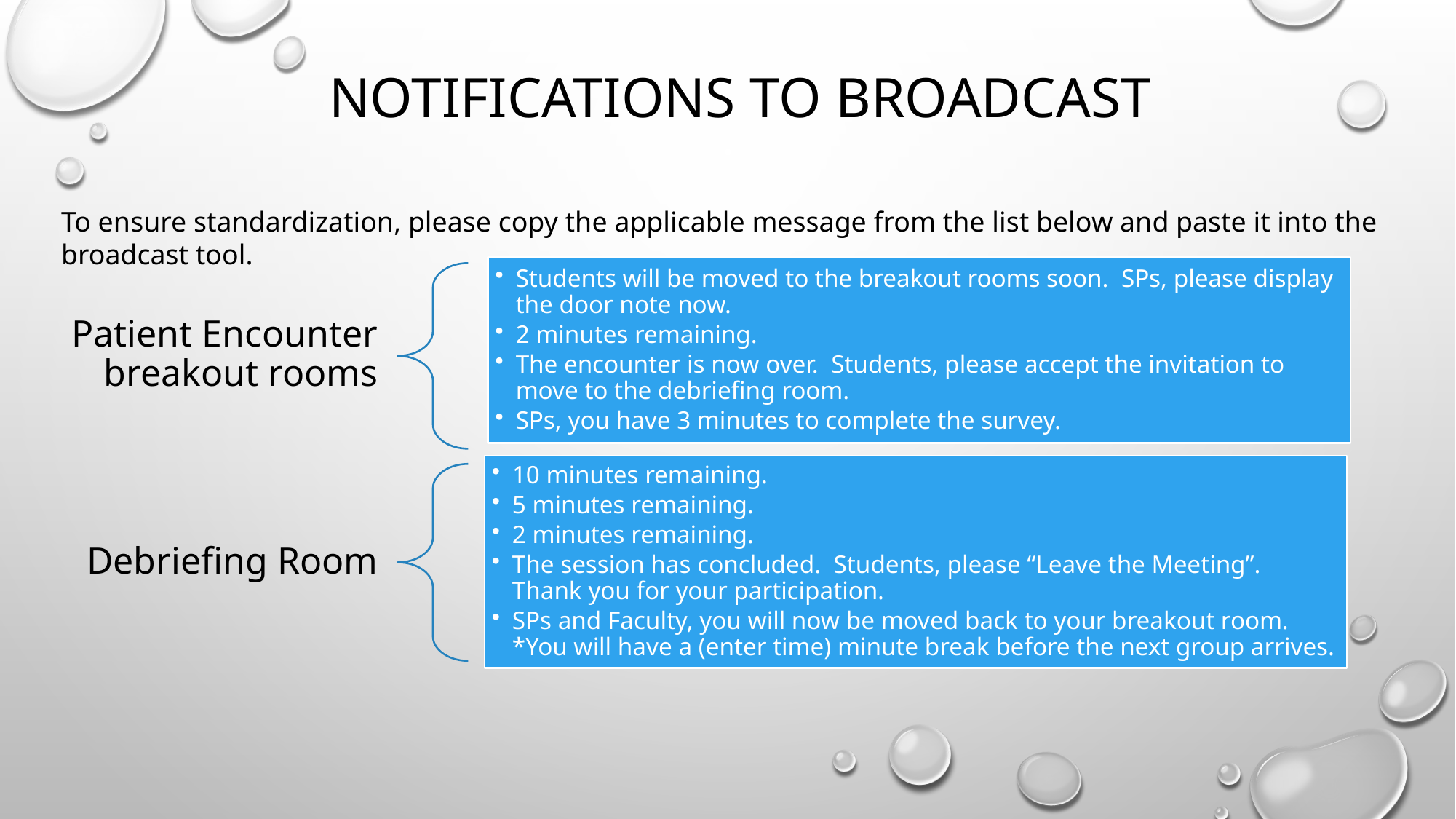

# notifications to broadcast
To ensure standardization, please copy the applicable message from the list below and paste it into the broadcast tool.
Students will be moved to the breakout rooms soon. SPs, please display the door note now.
2 minutes remaining.
The encounter is now over. Students, please accept the invitation to move to the debriefing room.
SPs, you have 3 minutes to complete the survey.
Patient Encounter breakout rooms
10 minutes remaining.
5 minutes remaining.
2 minutes remaining.
The session has concluded. Students, please “Leave the Meeting”. Thank you for your participation.
SPs and Faculty, you will now be moved back to your breakout room. *You will have a (enter time) minute break before the next group arrives.
Debriefing Room

## Slide 5
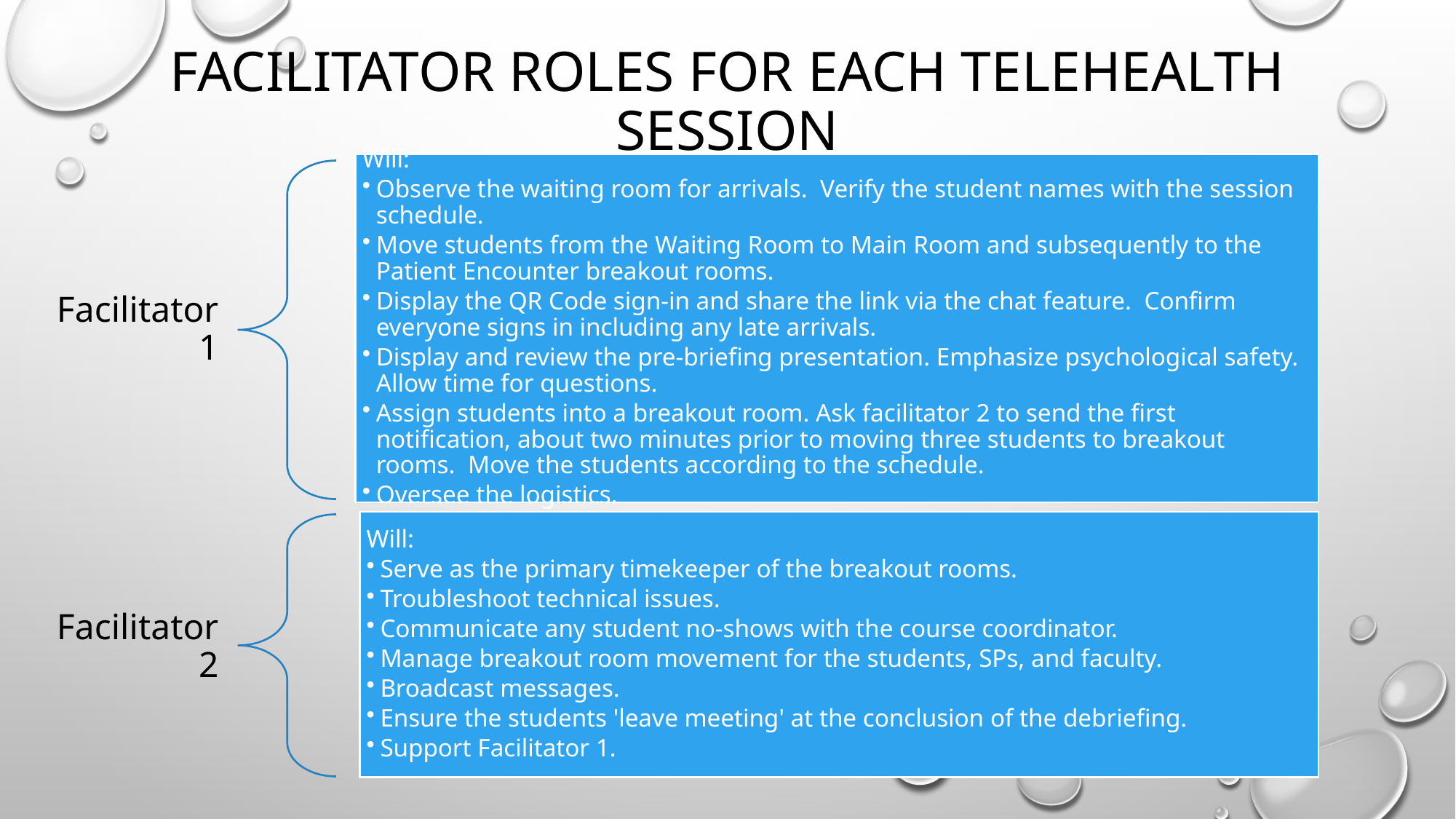

# Facilitator roles for each telehealth session
Will:
Observe the waiting room for arrivals. Verify the student names with the session schedule.
Move students from the Waiting Room to Main Room and subsequently to the Patient Encounter breakout rooms.
Display the QR Code sign-in and share the link via the chat feature.  Confirm everyone signs in including any late arrivals.
Display and review the pre-briefing presentation. Emphasize psychological safety. Allow time for questions.
Assign students into a breakout room. Ask facilitator 2 to send the first notification, about two minutes prior to moving three students to breakout rooms.  Move the students according to the schedule.
Oversee the logistics.
Facilitator 1
Will:
Serve as the primary timekeeper of the breakout rooms.
Troubleshoot technical issues.
Communicate any student no-shows with the course coordinator.
Manage breakout room movement for the students, SPs, and faculty.
Broadcast messages.
Ensure the students 'leave meeting' at the conclusion of the debriefing.
Support Facilitator 1.
Facilitator 2
