# Supplementary material for: Situational Awareness in Telehealth: A Virtual Standardized Patient Case for Transitioning Preclinical to Clinical Medical Students
Source: MedEdPORTAL. 2025 Apr 11;21:11517. doi: 10.15766/mep_2374-8265.11517 (PMC11985545; doi:10.15766/mep_2374-8265.11517)
Supplement: Supplementary file 1 — Student Prework.pptxFaculty Training Guide.docxSP Scenario.docxSP Survey Tool.docxScenario Stem.pptxStudent Prebriefing.pptxSession Facilitators Presentation.pptxPostencounter Student Survey.docx [file mep_2374-8265.11517-s001.zip › C. SP Scenario.docx]

Date: June 2020 – present

Primary Case Author: Alecia Sabartinelli Stein, MD

Secondary Case Author: Roxanna Araya

Standardized Patient Educator: Roxanna Araya

Name of Case: Melanie/Matthew Jones Telehealth Assessment

Name of Educational and/or Assessment Activity: Telehealth Module

Patient Name: Melanie or Matthew Jones

Chief Complaint: Palpitations

Most Likely Diagnosis and Differential with Rationale from History and/or Physical Exam:

Most likely Diagnosis:

Anxiety – Patient with history of uncontrolled anxiety, who is not able to afford prescribed anxiolytic medications, presents with palpitations worse during times of stress. No personal or family history of cardiac disease. No associated symptoms such as loss of consciousness.

Differential diagnosis:

Palpitation Complaint: Facial Lesion:

Cardiac Arrythmia Mole

Hyperthyroidism Benign growth

Medication side effects Malignancy (cancerous tumor, melanoma)

Anxiety Bruise, innocent or victim of physical abuse

Challenge Question: Does the learner ask the SP to remove his/her/their sunglasses?

Domains: Check all that apply

X Professionalism

X Communication and Interpersonal Skills

X Medical History

X Physical Exam

- Shared Decision-Making
- Patient Education

X Clinical Reasoning

- Documentation
- Handoff
- Presentation

X Other: Situational Awareness in telehealth

Type and Level of Learner: Pre-clinical second year medical students

Case Objectives:

By the end of this activity, learners should be able to:

1. Outline key components of a typical telehealth encounter.
2. List effective strategies to reduce communication errors in the telehealth clinical environment.
3. Demonstrate a focused patient assessment, including assurance of proper patient, setting, and confidentiality during a telehealth visit.
4. Explain the vital components of active situational awareness (SA) in the telehealth clinical environment.

| SETTING: outpatient, in patient, ED, home, nursing home, rehab, group, etc. | Virtual setting |
| --- | --- |
| PATIENT PROFILE: Information about the “patient” that helps select an SP and helps the learner get an understanding of them as a person. SP will know more information about the patient than learner will ever ask but allows SP to portray a fully developed patient personality. If none of the items below are particulars for the case, please write “all may be used.” | |
| Age range | Use your own, or choose an age between 22 to 70 years old |
| Religious/spiritual background | Any |
| Sex (e.g., male, female, intersex, transwoman, transman) | Use your own |
| Sexual orientation (e.g., heterosexual, lesbian, gay, bisexual, pansexual, queer, asexual) | Heterosexual |
| Gender expression (e.g., man, woman, genderqueer) | Use your own |
| Race and ethnicity (e.g., to promote educational diversity, we use a diverse pool of SPs.) | Use your own |
| Physical description (e.g., BMI, height range) | Use your own |
| Physical limitations | None |
| Patient appearance (e.g., disheveled, hospital gown, business casual, casual) | Casual, at home. Wearing dark sunglasses.  Facial lesion located on the right side of the face. The skin lesion should be visible below the rim of the sunglasses. |
| Moulage + location (e.g., none, bruises, scars, body piercing, tattoos) | Supplies:  Brown/mauve eye shadow pallet with applicator  Brown eye liner pencil  Dark sunglasses  Instructions:  Put on dark glasses. Make a few dots with brown eyeliner pencil 1-2 inches below the bottom rim of the glasses.  Remove the glasses and add dots an inch or two around the initial dots to create an uneven circular shape  Connect the dots.  Use tapping motions with the eyeshadow applicator (rather than spreading) to apply brown and mauve eye shadow to fill in the areas outlined with the pencil. Next, use a tapping method with the applicator to apply some gold-colored eye shadow on top.  Re-place glasses.  The lesion should be partially covered by the glasses with about half of the lesion peeking out below the bottom rim of the glasses.  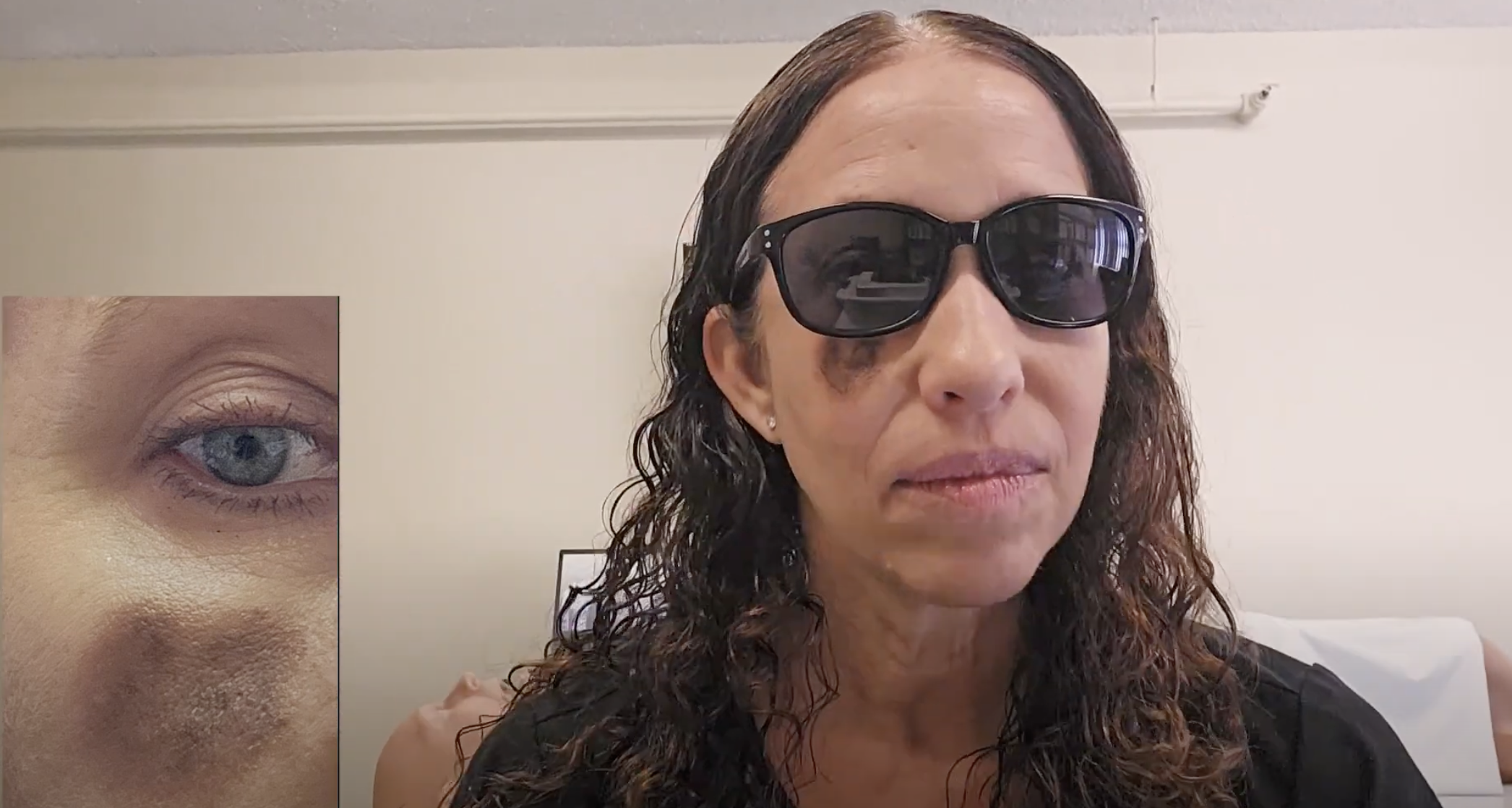  *Author-owned image* |
| Affect (e.g., pleasant, cooperative) | Concerned, slightly low, anxious.  If you are asked to remove your sunglasses, your body language should read that you are mildly embarrassed because of the facial lesion. |
| Family group (e.g., who is family, who they live with) | Lives alone, entire family lives out of state, single, broke up with partner 3 weeks ago |
| Education | Associates degree |
| Level of health literacy | Average |
| Employment, if any - present and past, noting any current stresses | Currently unemployed.  You were employed as a receptionist until 3 months ago. Due to company cutbacks, you were released from your job. |
| Home/homeless - type of dwelling, number of stories, owned or rented | Renting in Miami, single-story apartment |
| Financial situation - any current stresses | Unemployed as of 3 months, positive for financial stressors due to lack of paycheck, “I do feel stressed about not having a job and no health insurance. I need to refill my medications but can’t without insurance. I just can’t afford it right now.” |
| Insurance status (e.g., un/under/insured, public/private, HMO/PPO) | Uninsured due to unemployment |
| Habits (i.e., diet, exercise, caffeine, smoking, alcohol, drugs) | Regular diet, having to cook a lot at home lately.  Walking on occasion, it sometimes helps with stress.  Tobacco: ½ pack per day for 5 years.  Alcohol: On the weekends – 2-3 glasses of wine about twice a week.  Drugs: None |
| Activities (i.e., hobbies, sports, clubs, friends) | Hobbies include reading and watching the news. Not in any sports or club teams. Has local friends, but they are not good listeners. |
| Typical day - what is the usual daily routine | Just sitting around at home ever since losing job. |

| CASE INFORMATION | |
| --- | --- |
| Chief Concern: What the patient will say when greeted by the student. The patient’s primary reason for seeking medical care often stated in their own words. | I’ve been having heart palpitations for a couple of months, and they felt worse last night. *(Do not offer any additional information unless prompted/asked.)* |
| Additional Concerns: Other, if any, concerns the patient has today (i.e., symptoms, requests, expectations, etc.) that will become part of set agenda. | No medical insurance for the past two months and unable to refill prescription medications for about 6 weeks.  Moderate to severe stress due to unemployment, lack of insurance, lack of prescriptions, and general world issues going on (ongoing wars, rising rent in Miami). |
| THE PATIENT’S STORY: The SP will be asked to tell their symptom story and the personal and emotional impact for each of their concerns. You will want to write this in the patient’s voice. The symptom story should be able to answer this question: “Tell me more about [chief concern/additional concern], starting at the beginning and bringing me up to now.”  The personal context should be able to answer questions concerning the broader personal/psychosocial context of symptoms, especially the patient’s beliefs/attributions.  The emotional context should be able to ask how are you doing with this, how does this make you feel, how has this affected you emotionally? IMPACT: How has this affected your life? How has this been for your family? | I usually take deep breaths, sit down, or try to relax when I feel them. They usually go away pretty quickly.  Last night and this morning, however, I could feel my heart pounding and racing and coming back more frequently.  *If asked:* They seem to last about 5 minutes and are returning every few hours.  I’m worried something is wrong with my heart and that I won’t be able to afford my medical treatment since I no longer have insurance. I already haven’t been able to refill my medications after I lost my job.   Sunglasses related questions:   - I wear them because I feel more comfortable when they are on. - *If you are asked to remove the sunglasses, please remove them. Your body language should read that you are a little embarrassed*.   Facial lesion related questions:   - If asked about the lesion: Touch the area and reply that it has been there for a while. - Physical changes: It has gotten darker and bigger. - Sun exposure: As a teenager and a college student, I enjoyed tanning (a sun worshipper) and never used sunscreen. - Abuse related questions: I feel safe and have not suffered any kind of physical abuse or injury.   Faculty debriefing points may include:   - How did you feel during the encounter? What is your initial reaction(s)? What went well? What would have been done differently? - How did you establish rapport via a telehealth mode of interaction? - How did you confirm the correct patient? What is the best approach to set up for a telehealth visit (zoom background, quiet, well-lit space, well framed and face into the camera)? - How was the flow of information? Was communication clear? Did you use closed loop communication? - Did you notice anything else about the patient?   **SP reminder: Please wear your sunglasses when joining the debriefing breakout room. The faculty debriefer will ask all the SPs to remove their sunglasses at the designated time. |
| HISTORY OF PRESENT ILLNESS: Although some of the HPI will be given in the patient’s symptom story, the learners will expand the story during the direct question section. Below, describe the detailed history, usually about the chief concern, which the student must develop in order to make a useful assessment of the problem: | |
| Onset (when; gradual or sudden) | I have been having them on and off for the past few months (2-3 months). |
| Setting (what was going on or where was patient when symptoms first noticed?) | I first noticed this when I was going to sleep, I noticed it again in the morning when I woke up. |
| Duration (how long) | They usually last 2-3 minutes. Last night, and today, they feel more frequent. |
| Time relationships (frequency, constant or intermittent) | Intermittent |
| Location | My heart, I feel my heart beating inside my chest. |
| Radiation | No |
| Quality | They feel like butterflies. I do not get dizzy from them and have  never lost consciousness or fainted. |
| Amount | 3x/week |
| Aggravated by what | Stress |
| Relieved by what | I usually sit/lie down, take deep breaths, and try to relax. This has not worked well since last night. |
| Associated with what | They seem to get worse when I feel stressed.  They feel better when I try to focus on my breathing and relax. |
| Attitude (what does the patient think is the problem, and how do they feel about it) | I am worried something is wrong with my heart |
| Overall course | Progressed in severity. |
| REVIEW OF SYSTEMS: Significant positives and negatives – *this information must be elicited/asked by the student* | |
| Positives | Palpitations, anxiety, difficulty sleeping, loss of appetite |
| Negatives | Fever, SOB, chest pain, weight loss, urinary symptoms, fatigue, headache, dizziness, loss of consciousness |
|  |  |
| Past medical history | Overall normal |
| Medication allergies (name and reaction) | None |
| Environmental allergies (name and reaction) | None |
| Illnesses | None |
| Vaccinations | Up to date |
| Surgeries | Tonsillectomy at 8 yrs of age for recurrent strep infections |
| Accidents/injuries/trauma | None |
| Hospitalization | None |
|  | |
| Inclusive sexual and reproductive history | |
| Sexual practices  Sexual partners  Protection: Use of safer sex practices  Use of birth control if appropriate  Risk of intimate partner violence | Current: single, recently broke up with partner   \| Monogamous: \| 0 currently \| \| --- \| --- \| \| Number of partners: \| 3 \| \| Orientation: \| Heterosexual \| \| Form of birth control: \| Pill/condoms \|   Past:   \| History of STDs: \| No \| \| --- \| --- \| \| Tested for AIDS: \| No \| |
| OB/GYN history | *If female SP:*  Age of onset of menses: 12 years old  Age of menopause: N/A  Number of pregnancies: None  Number of live births: None  Number of miscarriages: None  Number of abortions: None |
| Medications | \| Over the counter: \| N/A \| \| --- \| --- \| \| Prescriptions: \| - Birth control (females only) - Anxiety Medication: Prescribed several years ago to control moderate anxiety symptoms.   - SP does not recall the name. \| |
| Immunizations | X Tetanus  X Flu  X Hepatitis  X Pneumovax  X HPV |
| Tobacco products:  X Cigarettes   - Cigar - Pipe - Chew - E-cigarettes | - Never - Past - year started/year quit   X Current   - - Quantity: ½ pack/day   - # of years: 5 years |
| Alcohol   - Beer   X Wine   - Liquor - Other | - Never - Past - year started/year quit   X Current   - - Quantity: 2-3 glasses of wine on the weekends   - # of years: 5 years |
| Drugs   - Weed - Cocaine - Heroin - Meth - IV - Inhalants - Other | X Never   - Past - year started/year quit - Current   - Quantity   - # of years |
| Diet (describe) | Pretty regular diet, having to cook a lot at home lately. |
| Exercise (describe) | Walking on occasion, it sometimes helps with stress. |
| List any other important social history or information important to this case | Loss of job 3 months ago. You used to get a lot of sun as a teenager as you grew up next to the beach. |
| Family history |  |
| Mother, father, siblings, grandparents, and other significant findings | Mother: alive, HTN  Father: alive, diabetes  Siblings: 1 sister, healthy  Grandparents: alive, both have diabetes and HTN |
|  |  |
| Physical Exam - List exam maneuvers expected for this case and any abnormal findings that SP will simulate. (tenderness, hyper-hypo reflex, rebound, weakness, etc.)  *****Virtual physical exam*****  -General appearance  -Skin: check for any new rashes, bruises, or swelling | |
| PHYSICAL EXAM FINDINGS |  |
| 1. Written in layperson’s terms | Anxious demeanor. Wearing dark sunglasses. Facial lesion located on the right side of the face. The skin lesion is slightly visible below the rim of the sunglasses. |
| 1. General appearance - affect, appearance, position of patient at opening (i.e., sitting, lying down, holding abdomen, etc.) | Sitting on a chair in front of computer. Well developed, well nourished, under no distress. Anxious affect. |
| 1. Vital signs | n/a (on zoom) |
| 1. Specific findings and affect | Anxious when speaking about symptoms and social circumstances. Feeling self-conscious about facial lesions and didn’t immediately want to take of sunglasses. |
| 1. Response to certain physical movements | n/a |
|  |  |
| DIAGNOSIS AND DIFFERENTIAL |  |
| Diagnosis with support from positive and negative history and PE findings | Anxiety: history of anxiety that has been uncontrolled ever since patient has not been able to afford medication. Palpitation have been worse with stress. No personal or family history of cardiac disease. Significant stressors recently. + New finding of a potential melanoma due to growing skin lesion on the face and excess sun exposure as a child. |
| Differential with support from positive and negative history and PE findings | Arrythmia: Palpitations, feeling fluttering of heart. However, no family history of early cardiac death. No associated symptoms such as loss of consciousness.  Victim of abuse: Given the patient's symptoms of anxiety and trying to conceal a facial lesion with sunglasses, this is a presentation that is worrisome for signs of abuse. The lesion looks more like a melanoma-like mole than a bruise, so this presentation is likely melanoma. However, the facial lesion was evaluated through a screen, thus the patient must still have an in-person follow-up to confirm the characteristics of the lesion. Since abuse is on the differential, it would not be incorrect to ask safety related questions during the encounter. |
|  |  |
| MANAGEMENT OR DIAGNOSTIC PLAN | - Follow up with dermatology regarding skin lesion. - Offer support for financial burden, discuss affordable insurance options. - Restart anxiety medication once the patient can afford to do so. |
|  |  |
| PROFESSIONALISM ISSUES OR CHALLENGES | Real time coordination of staff and learners within virtual rooms |
