# Supplementary material for: Situational Awareness in Telehealth: A Virtual Standardized Patient Case for Transitioning Preclinical to Clinical Medical Students
Source: MedEdPORTAL. 2025 Apr 11;21:11517. doi: 10.15766/mep_2374-8265.11517 (PMC11985545; doi:10.15766/mep_2374-8265.11517)
Supplement: Supplementary file 1 — Student Prework.pptxFaculty Training Guide.docxSP Scenario.docxSP Survey Tool.docxScenario Stem.pptxStudent Prebriefing.pptxSession Facilitators Presentation.pptxPostencounter Student Survey.docx [file mep_2374-8265.11517-s001.zip › D. SP Survey Tool.docx]

| **Simulated Patient Survey Tool** | | |
| --- | --- | --- |
| 1. Simulated Patient Name: |  | |
| 1. Did the student introduce himself / herself? | Yes | No |
| 1. Did the student confirm your name to ensure you are the correct patient? | Yes | No |
| 1. Were you asked about the facial lesion? | Yes | No |
| 1. Were you asked to remove your sunglasses? | Yes | No |
| 1. Did the student offer a follow up plan or possible next steps? | Yes | No |
| 1. Comments: | | |
