# Supplementary material for: Situational Awareness in Telehealth: A Virtual Standardized Patient Case for Transitioning Preclinical to Clinical Medical Students
Source: MedEdPORTAL. 2025 Apr 11;21:11517. doi: 10.15766/mep_2374-8265.11517 (PMC11985545; doi:10.15766/mep_2374-8265.11517)
Supplement: Supplementary file 1 — Student Prework.pptxFaculty Training Guide.docxSP Scenario.docxSP Survey Tool.docxScenario Stem.pptxStudent Prebriefing.pptxSession Facilitators Presentation.pptxPostencounter Student Survey.docx [file mep_2374-8265.11517-s001.zip › H. Postencounter Student Survey.docx]

1. The Telehealth patient encounter with a simulated patient was valuable.

| Strongly Agree | Agree | Disagree | Strongly Disagree | Not Applicable |
| --- | --- | --- | --- | --- |

1. I am able to identify key components of a typical telehealth patient encounter (i.e. introductions and patient verification) following the Telehealth Patient Encounter scenario.

| Strongly Agree | Agree | Disagree | Strongly Disagree | Not Applicable |
| --- | --- | --- | --- | --- |

1. As a result of this Telehealth Patient Encounter and faculty debriefing session, I realize the importance of maintaining active situational awareness during my future patient encounters.

| Strongly Agree | Agree | Disagree | Strongly Disagree | Not Applicable |
| --- | --- | --- | --- | --- |

1. I feel attuned to recognize and address patient-specific characteristics, mannerisms or appearances that may indicate a health-related issue. For example, I would notice a lesion and inquire about it or ask a patient wearing glasses to remove them.

| Strongly Agree | Agree | Disagree | Strongly Disagree | Not Applicable |
| --- | --- | --- | --- | --- |

1. I feel better prepared to address uncomfortable/awkward moments following the Telehealth Patient Encounter.

| Strongly Agree | Agree | Disagree | Strongly Disagree | Not Applicable |
| --- | --- | --- | --- | --- |

1. We welcome your feedback below.

|  |
| --- |
